# Demographic estimates from the Palaeolithic–Mesolithic boundary in Scandinavia: comparative benchmarks and novel insights

**DOI:** 10.1098/rstb.2020.0037

**Published:** 2020-11-30

**Authors:** Victor Lundström, Robin Peters, Felix Riede

**Affiliations:** 1 University Museum, University of Bergen, Villaveien 1A, 5020 Bergen, Norway; 2 LVR-State Service for Archaeological Heritage, Endenicher Strasse 133, 53115 Bonn, Germany; 3 Department of Archaeology and Heritage Studies, Aarhus University, Moesgård Allé 20, 8270 Højbjerg, Denmark; 4 BIOCHANGE—Centre for Biodiversity Dynamics in a Changing World, Aarhus University, Ny Munkegade 116, 8000 Aarhus C, Denmark

**Keywords:** population dynamics, hunter–fisher–gatherers, final Palaeolithic, early Mesolithic, Scandinavian prehistory

## Abstract

Prehistoric demography has recently risen to prominence as a potentially explanatory variable for episodes of cultural change as documented in the archaeological and ethnographic record. While this has resulted in a veritable boom in methodological developments seeking to address temporal changes in the relative size of prehistoric populations, little work has focused on the manner in which population dynamics manifests across a spatial dimension. Most recently, the so-called Cologne Protocol has led the way in this endeavour. However, strict requirements of raw-material exchange data as analytical inputs have prevented further applications of the protocol to regions outside of continental Europe. We apply an adjusted approach of the protocol that makes it transferable to cases in other parts of the world, while demonstrating its use by providing comparative benchmarks of previous research on the Late Glacial Final Palaeolithic of southern Scandinavia, and novel insights from the early Holocene pioneer colonization of coastal Norway. We demonstrate again that population size and densities remained fairly low throughout the Late Glacial, and well into the early Holocene. We suggest that such low population densities have played a significant role in shaping what may have been episodes of cultural loss, as well as potentially longer periods of only relatively minor degrees of cultural change.

This article is part of the theme issue ‘Cross-disciplinary approaches to prehistoric demography’.

## 1. Introduction

Numerous instances in the archaeological and ethnographic record point to the slow development [1], sudden introduction [2,3] or sudden disappearance [4,5], of specific cultural traits. How can we best explain these patterns? Part of the answer may lie in palaeodemography [6], and while its connection to societal or organismic change is not novel *per se* [7–9], it is within the field of cultural evolution [10] that these ideas have gained most traction. Cultural evolutionary theory views technological or cultural change as underwritten by an inheritance system akin to, while simultaneously different from, genetic inheritance [11], whereby information, perhaps the kind alluding to the creation or maintenance of a specific technological tool, is passed on between individuals over generations [12]. However, the social contexts where said transmission occurs will always vary in its ability to maintain the fidelity of the information that is being transmitted [13] resulting in transmission errors [14], which, in turn, gives rise to novel cultural traits [15].

Different inheritance pathways are not the only distinct features separating cultural from genetic transmission, however. Indeed, perhaps the most striking difference can be found in the elaboration by Strimling *et al.* [16, p.13 870] who suggests that while ‘genetic information is acquired only once, cultural information can be both abandoned and reacquired during an individual's lifetime’. This long-term process of acquiring and reacquiring culture, through various forms of social learning [17,18], has also led to the proposition that humans have a unique capacity to maintain cumulative culture [19] whereby a previous pool of knowledge in any given population may allow cultural traits to be adjusted, and perhaps even improved, incrementally rather than being invented or re-invented single-handedly. The reason demography has risen to prominence as a potentially explanatory variable for this phenomenon—apart from that it parallels the crucial link to biological change as reflected in gene frequencies [20]—is because while cumulative culture is more easily maintained in larger populations, owing to its potential for minimizing loss of knowledge in the event stochastic change [1], the opposite is expected for smaller and less connected populations [4].

The dynamic changes in a population's size and density are ultimately the summary outcome of decisions related to its life history that are cued, and in part determined, by climate and the environment [3,21,22]. In fact, the mere variability and amplitude of different ecological contexts in general, and their tendency to increase subsistence failure in particular, has generated a counter-proposal against the role of demography and its causal role in episodes of cultural change. Namely that hunter–gatherers have proclivity for technological experimentation in order to avoid returning to camp empty-handed [23]. Numerous tests that claim support for this hypothesis have followed [24–27]. However, incorporating ethnographic reference data has been suggested to invalidate these tests, partly because densities as recorded in the ethnographic record might be misleading [28, p. 140] but also because they might not accurately represent what Henrich [4] and Henrich *et al.* [29] refer to as the effective population size, or the knowledge-sharing portion of a population.

For instance, among the examples providing support for the role of demography and technological complexity, Powell *et al*. [2] demonstrated how symbolic and technological features, considered hall-marks of modern human behaviour, occurred together with increased population densities and migration rates as early as 45 000 years ago. In this example, the effective population size was emulated using genetic estimates on population size and areal estimates in km^2^ of Europe during the Pleistocene [2, p. 1301]. Similarly, Kline & Boyd [30] also reported a positive relationship in their study, where modern maritime communities in Oceania considered to be highly connected across space, had more tools than those considered less connected. However, framing the debate as two competing hypotheses runs the risk of establishing a dichotomy between demography and environment, whereas they might be linked by a form feedback [31]. For instance, while numerous and more sophisticated tool kits could lead to greater subsistence yields and concomitant population growth, the technological improvement in itself could potentially lead to environmental overexploitation [32], potentially creating or aggrevating long-term negative effects [33].

Demography has long been considered an important factor in discussions of technological change and social complexity in Scandinavian prehistory [34–36]. However, it is only within the last decades that researchers have highlighted the end of the Late Glacial and early post-glacial [37–41] as a series of colonization attempts, where both cultural and climatic factors triggered severe and repeated episodes of demographic collapse [42,43] with concomitant cultural and technological loss [5,44]. For instance, following Morin [45], Riede [37] summarized diversity estimates of mammalian prey species in southern Scandinavia during the Late Glacial Final Palaeolithic, treating ethnographic estimates on population density as a dependent variable of the former. With a strong correlation in the dataset, estimates were very low indeed (0.2–0.3 persons/100 km^2^) and aligned well with others obtained for the Late Glacial Final Palaeolithic [46,47].

Ethnographic accounts on population densities are not without problems, however, as the specific historical circumstances during their recording might have produced erroneous estimates ([45, p. 53]; [48]). Moreover, taphonomic processes [49] often prevent even the most basic inference on prehistoric subsistence practice, making it difficult to consistently model palaeodemographic estimates across different regions and time periods. Estimates from palaeogenetics are no different. While later colonization pulses into Scandinavia (9500–6000 cal BP) appear to demonstrate a fairly high effective population size [41], the lack of any type of human remains associated with earlier colonization attempts makes it difficult to assess whether such estimates can be uncritically extrapolated back in time [50].

Palaeodemographic estimates not only need to be explicit about the connectedness or density of a population—important if they are to be applied in studies of cultural change, but they also need to be comparable across space and time. Numerous ways of inferring the absolute size and densities of past populations have been put forward previously [40,51], but in recent years, the Cologne Protocol (*hereafter* CP) has seen a particularly wide application [47,52–54]. The CP derives the absolute size and densities of a given prehistoric time period by a mathematical up-scaling approach, using a combination of ethnographic reference data [55], geostatistical estimates on landscape areas of intense occupation (called ‘core areas’) as well as polygons representing spatially explicit reconstructions on raw-material sourcing (called ‘extended areas’, [53,56]). By dividing the km^2^ extent of extended areas with that of core areas, the CP obtains an estimate for the number of Binford's GROUP2 social units [55] within each core area, and should, therefore, reference groups that practice an all-year-round exploitation of any given region ([56]; electronic supplementary material).

However, detailed reconstructions of raw-material sourcing is, if not impossible, very difficult to obtain in many other regions [47,48,57], and transferring estimates to regions where such data are missing has been advised against [53]. Furthermore, the archaeological record of Late Glacial Final Palaeolithic and early post-glacial Scandinavia points to highly ephemeral occupations [44,50], making it unreasonable to reconstruct highly aggregated family units as in some parts of continental Europe. Thus, in this article, we apply a purely modelling-based adjustment of the CP [58] that provides considerably more leeway with regard to the above-mentioned short-comings of the original CP approach. We demonstrate its use by reporting comparative benchmarks and revised population estimates for the Late Glacial Final Palaeolithic (14 000–13 000 cal. BP) of southern Scandinavia, as well as novel demographic insights for the early Mesolithic (11 500–10 000 cal. BP) of Holocene coastal Norway. Together, they form two very important case studies, seeing as both represent the northward migrations into environs previously uninhabited by humans at a time when dramatic and substantial climatic changes occured [59].

## 2. Material and methods

We use georeferenced site locations from previously published material [47,60,61], where Late Glacial Final Palaeolithic sites (*n* = 197)—primarily on a typological basis—date to Greenland Interstadials 1d-b (*ca* 14 000–13 000 cal. BP), whereas early Holocene sites (*n* = 767) are dated by a combination of either ^14^C, shoreline chronology and lithics typology to *ca* 11 500–10 000 cal. BP. We replicate the geostatistical component of interpolating and delineating ‘core areas’ using R-studio v. 3.1.4. [62] using the script provided by Schmidt *et al*. [56]. Supporting data can be found in our electronic supplementary material [63]. Core areas are delineated by plotting successive isolines on a continuous raster surface of interpolated settlement densities, using Euclidean distance measures of the so-called Largest Empty Circles [64]. Each isoline is converted into area-specific polygons, and the isoline deemed representative for core areas—also known as the ‘optimally describing isoline’ (ODI)—is identified by a maximum increase of space per equidistance of site densities. We also select a second peak in areal growth [58], but, instead of referring to them as ‘extended areas’, we adopt the term ‘home range’ coined by Burt [65, p. 351] as ‘that area traversed by the individual in its normal activities of food gathering, mating and caring for young’.

To model the number of social units, we assume groups to take part in a fission-and-fusion cycle [66,67]. Groups that map onto this kind of dynamic are best referenced by Binford's GROUP1 social units that disperse during parts of their settlement cycle [55]. To derive the number of groups, we divide the median km^2^ of home ranges with the km^2^ of the core areas (see table 1 for a summary). Population ranges are obtained by multiplying the number of groups with the maximum, 75th percentile, median, 25th percentile and the minimum estimates of group size from 16, primarily terrestrially oriented, ethnographic reference groups [46,47] that we hold constant across both case studies. While no osteological remains from early Holocene Norway can corroborate a fully marine economy, ample technological evidence suggest degrees of cultural inertia from the Late Glacial Final Palaeolithic to the early Holocene [68,69]. A marine economy is not evident until the late Preboreal [70], but to control for possible taphonomic distortion during its earlier stage, we provide comparative estimates using nine marine oriented reference groups in our electronic supplementary material, figure S7. Both sets of reference groups are calculated using both GROUP2 and GROUP1 social units for comparison (electronic supplementary material, figure S8).

**Table 1.** Summary of demographic parameters obtained, and the formulas or mode for calculating them.

protocol outputs	abbreviations	mode of calculation
core area in km^2^	Aca	ordinary kriging and first peak ODI
median home range in km^2^	Mhr	ordinary kriging and second peak ODI
number of groups	Ng	Ahr/Aca
group size	Gs	max, 75th percentile, median, 25th percentile, and min
number of people	Np	max, 75th percentile, median, 25th percentile, and min * Ng
density within core areas	Dca	Np/Aca
density within home ranges	Dhr	Np/Ahr
metapopulation density	Dmp	Np/Atac
total area of calculation in km^2^	Atac	polygons of modern national borders

## 3. Results

Our palaeodemographic estimates, summarized in table 2, are based on an ODI for core areas and home ranges at a 13.5 and 20 km equidistance for the Late Glacial Final Palaeolithic of southern Scandinavia, and 8.5 and 12.5 km equidistance for early Holocene Norway. In southern Scandinavia, a median population size of 432 people is bracketed by 201 (minimum) and 662 (maximum). Core area population densities are low (0.02–0.05 people/km^2^), but slightly higher across home ranges (0.09–0.28 people/km^2^). Lowest are metapopulation densities, hovering just above zero (0.002–0.006 people/km^2^). For early Holocene of Norway, a total median population size of 1159 people are followed by 541 (minimum) and 1777 (maximum). Both population densities within core areas (0.03–0.10 people/km^2^) and home ranges (0.10–0.32 people/km^2^) are somewhat higher. However, much like in southern Scandinavia, these territories are floating in much wider landscapes of significantly lower metapopulation densities (0.004–0.012 people/km^2^). A significant difference in the number of sites analysed in the two datasets, as well as the size of their demographic estimates is notable, and while it remains to be assessed if the former is a result of taphonomic distortion [71,72], the latter might very well be expected owing to improved climatic conditions of the Holocene [59, p. 556].

**Table 2.** Main results for southern Scandinavia and Norway (italics). (Mhr (median home range in km^2^), Tca (total km^2^ of core areas per region), Ng (number of GROUP1 social units), *R* (range), Gs (size of GROUP1 social units), Np (total number of people), Dca (population density within core areas), Dhr (population density within home ranges), Atac (km^2^ of total area of calculation), Dmp (metapopulation density). DK (Denmark), S Swe (southernmost Sweden), S Scand (southern Scandinavia), SE, N, C and SW Nor (southeastern, northern, central and southwestern Norway).)

	region	Mhr	Tca	Ng	R	Gs	Np	Dca	Dhr	Atac	Dmp
	DK	2369	12 244	16	max	23	368	0.03	0.16		0.003
					*Q*3	18	286	0.02	0.12		0.002
					median	15	240	0.02	0.10	54 496	0.002
					*Q*1	13	208	0.02	0.09		0.002
					min	7	112	0.01	0.05		0.001
	S Swe	2369	1178	13	max	23	299	0.25	0.12		0.002
					*Q*3	18	232	0.19	0.10		0.002
					median	15	195	0.16	0.08	54 496	0.002
					*Q*1	13	169	0.14	0.07		0.001
					min	7	91	0.08	0.04		0.001
total	S Scand	2369	13 422	29	max	23	667	0.05	0.28		0.006
					*Q*3	18	518	0.04	0.22		0.004
					median	15	435	0.03	0.18	54 496	0.004
					Q1	13	377	0.03	0.16		0.003
					min	7	203	0.02	0.09		0.002
	**region**	**Ahr**	**Tca**	**Ng**	**R**	**Gs**	**Np**	**Dca**	**Dhr**	**Atac**	**Dmp**
	*SE Nor*	*990*	*2261*	*7*	*max*	*23*	*153*	*0.07*	*0.15*		*0.001*
					*Q3*	*19*	*126*	*0.06*	*0.13*		*0.001*
					*median*	*15*	*100*	*0.04*	*0.10*	146 624	*0.001*
					*Q1*	*13*	*87*	*0.04*	*0.09*		*0.001*
					*Min*	*7*	*47*	*0.02*	*0.05*		*0.000*
	*N Nor*	*246*	*5721*	*20*	*max*	*23*	*459*	*0.08*	*1.87*		*0.003*
					*Q3*	*19*	*379*	*0.07*	*1.54*		*0.003*
					*median*	*15*	*299*	*0.05*	*1.22*	146 624	*0.002*
					*Q1*	*13*	*259*	*0.05*	*1.05*		*0.002*
					*min*	*7*	*140*	*0.02*	*0.57*		*0.001*
	*C Nor*	*2128*	*5153*	*14*	*max*	*23*	*331*	*0.06*	*0.16*		*0.002*
					*Q3*	*19*	*274*	*0.05*	*0.13*		*0.002*
					*median*	*15*	*216*	*0.04*	*0.10*	146 624	*0.001*
					*Q1*	*13*	*187*	*0.04*	*0.09*		*0.001*
					*min*	*7*	*101*	*0.02*	*0.05*		*0.001*
	*SW Nor*	*2128*	*4261*	*36*	*max*	*23*	*834*	*0.20*	*0.39*		*0.006*
					*Q3*	*19*	*689*	*0.16*	*0.32*		*0.005*
					*median*	*15*	*544*	*0.13*	*0.26*	146 624	*0.004*
					*Q1*	*13*	*471*	*0.11*	*0.22*		*0.003*
					*min*	*7*	*254*	*0.06*	*0.12*		*0.002*
	**Region**	**Ahr**	**Tca**	**Ng**	***R***	**Gs**	**Np**	**Dca**	**Dhr**	**Atac**	**Dmp**
*total*	*Norway*	*5492*	17 396	*77*	*max*	*23*	*1777*	*0.10*	*0.32*		*0.012*
					*Q3*	*19*	*1468*	*0.08*	*0.27*		*0.010*
					*median*	*15*	*1159*	*0.07*	*0.21*	146 624	*0.008*
					*Q1*	*13*	*1004*	*0.06*	*0.18*		*0.007*
					*min*	*7*	*541*	*0.03*	*0.10*		*0.004*

There is also a possibility that we selected too small of an ODI for early Holocene core areas, thus allowing differences to have become too pronounced. However, besides representing the maximum increase of space as indicated by our geostatistical analysis, an 8.5 km ODI for core areas is in fairly good agreement with foraging radii documented among coastal communities on the Northwest coast of America [73]. Moreover, most sites from early Holocene Norway point to a consistent pattern of being in close proximity to areas of high marine productivity and high degrees of mammalian diversity [74]. Thus, reduced foraging radii and larger populations, as a result of higher foraging returns per unit of time travelled may have been possible ([56]; electronic supplementary material).

## 4. Discussion

### (a) The Late Glacial: growth, stasis or thinning out

While some have suggested the Late Glacial to have been a period of population growth [51], others have suggested it to have been a period of relative population stability [75]. Continental-scale stability does not, however, preclude regional fluctuation. Kretschmer [46], for instance, calculated population levels and densities for the Late Pleistocene Hamburgian culture of Northern Europe that would have been at or below demographic viability, perhaps owing to a mobility-demanding subsistence strategy of reindeer hunting on an all-year-round basis [76, p. 133]. While these initial colonization attempts ultimately appear to have been demographically futile [42], our estimates demonstrate that the later migration pulse reflects a slight population growth. However, despite climatic improvements [44, p. 84] and the availability of markedly more diverse prey species in southern Scandinavia [37, p. 314], population estimates are still comparatively low, lending further support to previous modelling efforts [37], as well as reconstructions based on domestic group size derived from onsite data [77, p. 323]

**Figure 1.**
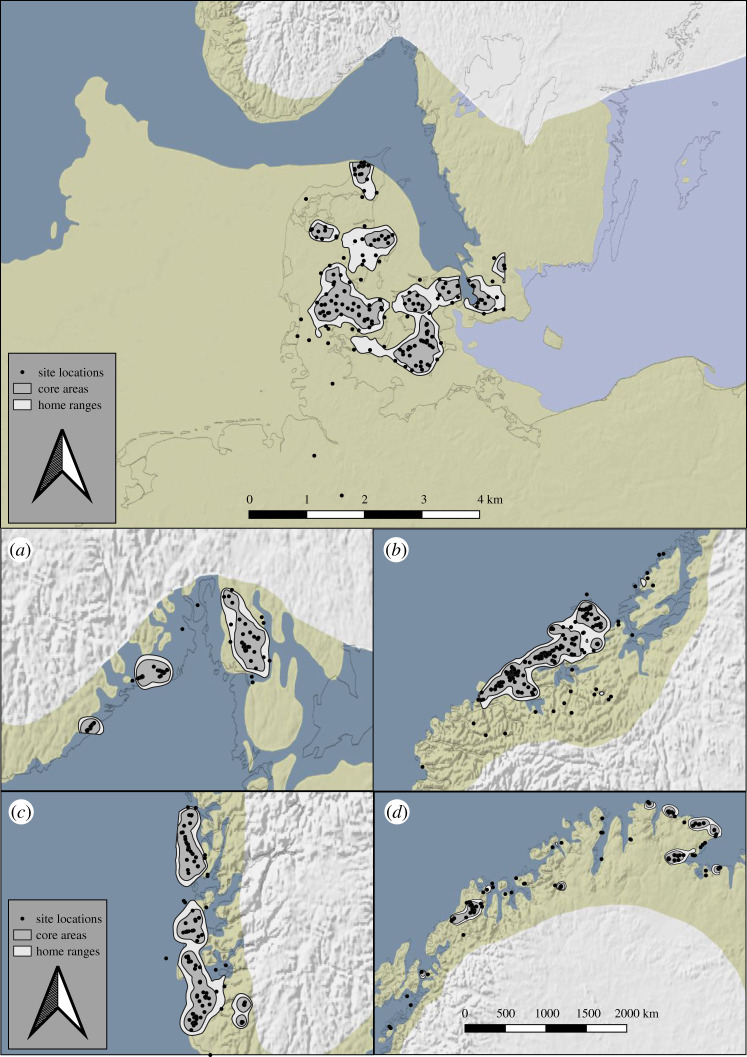
Distribution maps of site locations, core areas and home ranges, from southern Scandinavia during the Late Glacial Final Palaeolithic (top panel) and southeastern (*a*), central (*b*), southwestern (*c*) and northern Norway (*d*) during the early Holocene (bottom panel). Palaeogeographic maps were compiled by ZBSA after [78–91]. See the electronic supplementary material for a full literature list. Projection: UTM32N; EPSG: 25832.

Owing to the spatial explicitness of the CP, we can also address demographic estimates on a more local scale. For instance, although southernmost Sweden (Scania) potentially constituted a range contraction for Late Glacial populations (89–293 people), seeing as mainland Denmark holds the largest regional population size (112–369 people), the core area around lake Finja in northern Scania (figure 1, upper panel) constituted the largest median core area population size (154 people), even if only two out of four site locations in this area represent excavated sites. However, topographical conditions at the lake may have allowed for potential mass drives where flocks of reindeer could have been hunted in large numbers [92]. This would meet the expectation that, once resources become more predictable, they may potentially reduce the areal requirement of a group's territory, supporting in turn a larger population ([56]; electronic supplementary material).

Sparse numbers of excavated sites remain a common problem for the Late Glacial Final Palaeolithic. A majority of excavated sites are concentrated in southeastern Denmark, where both core areas and home ranges are largest and with the smallest populations (figure 1). Based on analyses of soil composition, Mortensen *et al.* [93, p. 203] conclude that a landscape of mostly birch woods would have allowed for diverse resource exploitation in the region. Interestingly, our geostatistical estimates appear to contradict this view. With such low population densities across core areas (0.02–0.05) and home ranges (0.09–0.28), the only option available to maintain any degree of viable bio-social reproduction would have been to increasingly rely on residential mobility strategies [94]. Accordingly, we would instead expect repeated and perhaps long-distance relocations of residential camps to have been a common strategy contributing, in turn, to the low population figures estimated for this region.

It is of course difficult to say at what exact population levels such mobility strategies would have been implemented. For instance, in a model of forager viability by White [95, p. 17] ‘as few as 75 persons would have a good chance of long term survival’, although Boone [96] also points at inherent instabilities of forager populations even in the absence of environmental forcing factors. Population crashes and local extinctions of forager populations are known both from ethnographic and ethno-historic records (see [97–100]), and once an already small population starts to decline in size, it could fall victim to a so-called extinction vortex where ‘processes such as environmental stochasticity, inbreeding, and behavioural failures' [101, p. 51] lead to population extinctions. Apart from the bio-social collapse that this would entail, repeated and severe collapses are also suggested to result in significant negative consequences for a population's ability to maintain any degree of cumulative culture [102]. Therefore, we consider such low levels in both population size and density as important components that helped structure an archaeological record that currently testifies to a highly limited duration of the respective technocomplexes associated with this region and time period (*ca* 14 000 to 13 000 cal. BP), as indicated by the relatively sparse radiocarbon record and only minor changes in the material culture [42,103].

### (b) Early Holocene Norway

Unambiguous traces of human occupation along coastal Norway do not appear until after the onset of the early Holocene [104,105] and especially after the receding fennoscandian ice-sheets facilitated safe passage across the Oslo fjord [106] from the Swedish west coast, where numerous and slightly older coastal locations are known [107–109]. Relative estimates from summed probability distributions of ^14^C-dates from northern Norway [110], as well as multiproxy reconstructions for southeastern Norway [111], provide the only comparative baseline for our estimates. However, as relative estimates cannot be translated into absolute number of people, our interpretations and comparisons will only be in the most tentative form. Somewhat counterintuitively, the region around the Oslo fjord (figure 1, bottom panel)—the region closest to the presumed source population in Sweden—would have been home to the smallest population for our entire study area (47–153 people), followed by the second largest population (140–459 people) in northern Norway. At its maximum population size, populations in southeastern Norway end up just above a so-called viability threshold [112], whereas conditions would have been more stable in the northern parts.

Lack of ^14^C-dates from southeastern Norway could potentially lend support to our estimates; however, Solheim & Persson [111] caution that a complete absence of ^14^C-dates might result from either taphonomic distortion, cultural practices that left little or no carbonized remains, or survey intensity, seeing as 10% of their sample represent sites dated to the early Mesolithic by other means. We have not compared our sample to that of Solheim & Persson [111], and thus, it is not clear if our demographic estimates from this region are simply skewed towards low population figures as a result of sample size. However, Jørgensen [110], with reference to pit dwellings excavated in northern Norway [113], suggest that early Mesolithic activity might be under-represented. Nonetheless, the demographic activity, although comparatively low to later time periods, appears to have endured only minor fluctuations in northern Norway. Perhaps such stability, combined with the beneficial effects that mixing ocean currents would have on local climatic conditions [110], helped increase the minimum–maximum range in population size as suggested in our estimates.

Potential episodes of stability might have been a common feature in this period. For instance, in central Norway, and apart from representing the third largest population (101–331 people), accumulation of sites appear stable over time [114]. More importantly, and in contrast with our earlier Late Glacial case study where extreme events might have had negative downstream effects on the cultural repertoire [5], there appears to have been no notable and negative effect on the technological composition for populations living in central Norway as a result of the Preboreal climatic event (9300–9200 BC), which seems to have lowered air and sea temperatures, re-expanded glaciers, thinned out of vegetational communities as well as prolonged seasonal ice-cover of nearby water bodies [74].

Although the following elaboration would require further testing, perhaps a flexible settlement strategy [60] could have helped to stimulate higher degrees of intergroup contact across home ranges, which for all coastal regions are among the densest in our model (0.10–0.32 people/km^2^) as opposed to potentially lower rates of intragroup contact within core areas (0.03–0.10 people/km^2^).

A predominantly coastal-oriented colonization of Norway finds support in our estimates so far, even if southwestern Norway provides a slight contrast. Most core areas are located at the outer archipelago (figure 1, bottom panel), occasionally along narrow straits where tidal currents generate conditions for high marine productivity [115]. However, two core areas are situated in the alpine areas to the southeast (figure 1, bottom panel). Located at less than or equal to 760 m above sea level, Bang-Andersen [116, p.112] interprets most sites in this region as seasonal camps, perhaps for specialized reindeer hunting. Nonetheless, southwestern Norway provide neither previous, nor chronologically overlapping, estimates for the early Mesolithic, thus we can assign little credibility to figures suggesting it to have held the largest population across the entire Norwegian coast (254–834 people). Future work is obviously needed and should preferably juxtapose our estimates with independent and additional proxies that relate to population size [117].

By combining ethnographic reference data and artefact distributions, Olsen & Alsaker [118] estimated a maximum population size of 558, and a minimum of 114 people, with a minimum population density of 55 people/km^2^ and a maximum of 270 people/km^2^ for the middle Mesolithic to Neolithic interval. Judging by our estimates, which are higher, this could suggest the presence of a potential inclination point towards population stasis at the end of the early Mesolithic.

The Storegga tsunami [119] could naturally have acted as a potential density-independent regulator [120, p. 3] for later time periods; however, our comparative estimates using more marine-oriented reference groups (electronic supplementary material, figure S7) also indicate that, aside from very high levels in northern Norway, a fully marine-based economy would have had a limited effect on the population size at the end of the Preboreal [70].

## 5. Conclusion

The causal role of demography in the cultural evolutionary process is debated intensely [121]. Various archaeological proxies can shed light on past demography, but such estimates must address both density and connectedness in order to be compatible with the foundational model of Henrich [4]. The CP has seen particularly wide application recently in providing such estimates, but requires, in its original formulation, raw material sourcing information across the entire study area in question. Such data are sparse, however, in many parts of the world, setting a barrier for further applications of the protocol. We have picked up on previous improvements to the protocol [58], yet our methodological contribution is modest at best. Using two important case studies of Scandinavian prehistory, we demonstrate the utility of the modified protocol outlined here by providing comparative benchmarks from the Late Glacial Final Palaeolithic, while breaking new ground with novel estimates from the early Holocene of coastal Norway. We demonstrate again that population size and densities remained fairly low throughout the Late Glacial, and well into the early Holocene. We suggest that such low population densities have played a significant role in shaping what may have been episodes of cultural loss, as well as potentially longer periods of only relatively minor degrees of cultural change. Future work should cross-check our estimates with other potential proxies that might relate to absolute estimates of both population size and density.

## Supplementary Material

Supplementary information and figures

## Data accessibility

Original data and results for the study can be found at https://github.com/VicluUiB/Demographic-estimates-from-the-Palaeolithic-Mesolithic-boundary_PhilTransB2020. The R-markdown for performing the geostatistical component of the protocol is accessed at https://github.com/C-C-A-A/CologneProtocol-R. More information is available in the electronic supplementary material.

## References

[1] Shennan S 2001 Demography and cultural innovation: a model and its implications for the emergence of modern human culture. Camb. Archaeol. J. 11, 5–16. (10.1017/S0959774301000014)

[2] Powell A, Shennan S, Thomas M 2009 Late Pleistocene demography and the appearance of modern human behavior. Science 324, 1298–1301. (10.1126/science.1170165) 19498164

[3] Kavanagh P, Vilela B, Haynie H, Tuff T, Lima-Ribeiro M, Gray R, Botero C, Gavin M 2018 Hindcasting global population densities reveals forces enabling the origin of agriculture. Nat. Hum. Behav. 2, 478–484. (10.1038/s41562-018-0358-8) 31097799

[4] Henrich J 2004 Demography and cultural evolution: how adaptive cultural processes can produce maladaptive losses—the Tasmanian case. Am. Antiq. 69, 197–214. (10.2307/4128416)

[5] Riede F 2009 The loss and re-introduction of bow-and-arrow technology: a case study from the Northern European Late Paleolithic. Lithic Technol. 34, 27–45. (10.1080/01977261.2009.11721072)

[6] Chamberlain A 2009 Archaeological demography. Hum. Biol. 81, 275–286. (10.3378/027.081.0309) 19943747

[7] Malthus TR 2003 An essay on the principle of population: text, sources and background, criticism, 2nd edn New York, NY: W. W. Norton.

[8] Boserup E 1965 The conditions of agricultural growth: the economics of agrarian change under population pressure. New York, NY: Aldine.

[9] Darwin C 1968 [1902] The autobiography of Charles Darwin (ed. F Darwin) New York, NY: Dover.

[10] Cavalli-Sforza L, Feldman M 1981 Cultural transmission and evolution. Princeton, NJ: Princeton University Press.

[11] Shennan S 2011 Descent with modification and the archaeological record. Phil. Trans. R. Soc. B 366, 1070–1079. (10.1098/rstb.2010.0380) 21357229 PMC3049110

[12] Tehrani J, Riede F 2008 Towards an archaeology of pedagogy: learning, teaching and the generation of material culture traditions. World Archaeol. 40, 316–331. (10.1080/00438240802261267)

[13] Apel J, Darmark K 2009 Evolution and material culture. Curr. Swedish Archaeol. 17, 11–28.

[14] Eerkens J, Lipo C 2005 Cultural transmission, copying errors, and the generation of variation in material culture and the archaeological record. J. Anthropol. Archaeol. 24, 316–334. (10.1016/j.jaa.2005.08.001)

[15] Lyman R, O'Brien M 1998 The goals of evolutionary archaeology: history and explanation. Curr. Anthropol. 39, 615–652. (10.1086/204786)

[16] Strimling P, Enquist M, Eriksson K 2009 Repeated learning makes cultural evolution unique. Proc. Natl Acad. Sci. USA 106, 13 870–13 874. (10.1073/pnas.0903180106) PMC2728987 19666615

[17] Boyd R, Richerson PJ 1985 Culture and the evolutionary process. Chicago, IL: University of Chicago Press.

[18] Richerson P, Boyd R 2005 The origin and evolution of cultures. Oxford, UK: Oxford University Press.

[19] Mesoudi A, Thornton A 2018 What is cumulative cultural evolution? Proc. R. Soc. B 285, 20180712 (10.1098/rspb.2018.0712) PMC6015846 29899071

[20] Metcalf CJE, Pavard S 2007 Why evolutionary biologists should be demographers. Trends Ecol. Evol. 22, 205–212. (10.1016/j.tree.2006.12.001) 17174004

[21] Codding B, Jones T 2013 Environmental productivity predicts migration, demographic, and linguistic patterns in prehistoric California. Proc. Natl Acad. Sci. USA 110, 14 569–14 573. (10.1073/pnas.1302008110) PMC3767520 23959871

[22] Tremayne A, Winterhalder B 2017 Large mammal biomass predicts the changing distribution of hunter-gatherer settlements in mid-late Holocene Alaska. J. Anthropol. Archaeol. 45, 81–97. (10.1016/j.jaa.2016.11.006)

[23] Torrence R 1989 Re-tooling: towards a behavioural theory of stone tools. In Time, energy and stone tools (ed. Torrence R), pp. 57–66. Cambridge, UK: Cambridge University Press.

[24] Collard M, Kemery M, Banks S 2005 Causes of toolkit variation among hunter-gatherers: a test of four competing hypotheses. J. Can. Archaeol. 29, 1–19.

[25] Collard M, Buchanan B, O'Brien M, Scholnick J 2013 Risk, mobility or population size? Drivers of technological richness among contact-period western North American hunter–gatherers. Phil. Trans. R. Soc. B 368, 20120412 (10.1098/rstb.2012.0412) 24101622 PMC4027409

[26] Collard M, Buchanan B, O'Brien M 2013 Population size as an explanation for patterns in the Paleolithic archaeological record. Curr. Anthropol. 54, S388–S396. (10.1086/673881)

[27] Buchanan B, O'Brien M, Collard M 2015 Drivers of technological richness in prehistoric Texas: an archaeological test of the population size and environmental risk hypotheses. Archaeol. Anthropol. Sci. 8, 625–634. (10.1007/s12520-015-0245-4)

[28] Boyd R, Richerson PJ, Henrich J 2013 The cultural evolution of technology: facts and theories. In Cultural evolution in society, technology, language and religion (eds Richerson PJ, Christiansen M), pp. 119–142. Strunmann forum reports 12 Cambridge, MA: The MIT Press.

[29] Henrich J, Boyd R, Derex M, Kline M, Mesoudi A, Muthukrishna M, Powell A, Shennan S, Thomas M 2016 Understanding cumulative cultural evolution. Proc. Natl Acad. Sci. USA 113, E6724–E6725. (10.1073/pnas.1610005113) 27791123 PMC5098628

[30] Kline M, Boyd R 2010 Population size predicts technological complexity in Oceania. Proc. R. Soc. B 277, 2559–2564. (10.1098/rspb.2010.0452) PMC2894932 20392733

[31] Shennan S 2015 Demography and cultural evolution. In Emerging trends in the social and behavioral sciences: an interdisciplinary, searchable, and linkable resource (eds Scott RA, Kosslyn SM), pp. 1–14. New York, NY: Sage Publications.

[32] Richerson P, Boyd R, Bettinger R 2009 Cultural innovations and demographic change. Hum. Biol. 81, 211–235. (10.3378/027.081.0306) 19943744

[33] Ghirlanda S, Enquist M, Perc M 2010 Sustainability of culture-driven population dynamics. Theor. Popul. Biol. 77, 181–188. (10.1016/j.tpb.2010.01.004) 20117125

[34] Petersen EB, Meiklejohn C 2007 Historical context of the term ‘complexity’. Acta Archaeol. 78, 181–192. (10.1111/j.1600-0390.2007.00105.x)

[35] Welinder S 1979 Prehistoric demography, Acta archaeologica Lundensia. Series tertia in 8° minore: LiberLäromedel/Gleerup: Habelt, *Lund: Bonn.*

[36] Price TD 1999 Human population in Europe during the Mesolithic. In Den Bogen spannen: Festschrift fur Bernhard Gramsch (eds Cziesla E, Kersting T, Pratsch S), pp. 185–195. Weissbach, Germany: Beier & Beran.

[37] Riede F 2009 Climate and demography in early prehistory: using calibrated ^14^C dates as population proxies. Hum. Biol. 81, 309–337. (10.3378/027.081.0311) 19943749

[38] Riede F et al. 2009 Tracking Mesolithic demography in time and space and its implications for explanations of culture change. In Chronology and evolution in the Mesolithic of N(W) Europe (eds Crombé P, Van Strydonck M, Sergant J), pp. 181–199. Newcastle, UK: Cambridge Scholars.

[39] Apel J, Wallin P, Storå J, Possnert G 2018 Early Holocene human population events on the island of Gotland in the Baltic Sea (9200–3800 cal. BP). Quat. Int. 465, 276–286. (10.1016/j.quaint.2017.03.044)

[40] Tallavaara M, Luoto M, Korhonen N, Järvinen H, Seppä H 2015 Human population dynamics in Europe over the Last Glacial Maximum. Proc. Natl Acad. Sci. USA 112, 8232–8237. (10.1073/pnas.1503784112) 26100880 PMC4500234

[41] Günther T et al 2018 Population genomics of Mesolithic Scandinavia: investigating early postglacial migration routes and high-latitude adaptation. PLoS Biol. 16, e2003703 (10.1371/journal.pbio.2003703) 29315301 PMC5760011

[42] Riede F, Pedersen JB 2018 Late Glacial human dispersals in northern Europe and disequilibrium dynamics. Hum. Ecol. 46, 621–632. (10.1007/s10745-017-9964-8)

[43] Riede F 2014 Success and failure during the Late Glacial pioneer human re-colonisation of southern Scandinavia. In Lateglacial and postglacial pioneers in Northern Europe, British archaeological reports (international series) 2599 (eds Riede F, Tallavaara M), pp. 33–52. Oxford, UK: Archaeopress.

[44] Riede F 2017 Splendid isolation: the eruption of the laacher See volcano and southern Scandinavian Late Glacial hunter-gatherers. Aarhus, Denmark: Aarhus University Press.

[45] Morin E 2008 Evidence for declines in human population densities during the early Upper Paleolithic in western Europe. Proc. Natl Acad. Sci. USA 105, 48–53. (10.1073/pnas.0709372104) 18172204 PMC2224228

[46] Kretschmer I, 2015 Demographische Untersuchungen zu Bevölkerungsdichten, Mobilität und Landnutzungsmustern im späten Jungpaläolithikum, Kölner Studien zur prähistorischen Archäologie. VML Verlag Marie Leidorf GmbH, Rahden/Westf.

[47] Lundström V, Riede F 2019 A spatially explicit model of Final Palaeolithic population densities for southern Scandinavia in the period between 14 000 and 12 700 cal BP. J. Archaeol. Sci. Rep. 26, 101886 (10.1016/j.jasrep.2019.101886)

[48] Maier A 2017 Population and settlement dynamics from the Gravettian to the Magdalenian. Mitteilungen Der Gesellschaft Für Urgeschichte 26, 83–101.

[49] Lyman RL 1987 Zooarchaeology and taphonomy: a general consideration. J. Ethnobiol. 7, 93–117.

[50] Bjerck HB 2008 Norwegian Mesolithic trends: a review. In Mesolithic Europe (eds Bailey G, Spikins P), pp. 60–106. Cambridge, UK: Cambridge University Press.

[51] Bocquet-Appel J, Demars P, Noiret L, Dobrowsky D 2005 Estimates of Upper Palaeolithic meta-population size in Europe from archaeological data. J. Archaeol. Sci. 32, 1656–1668. (10.1016/j.jas.2005.05.006)

[52] Maier A, Zimmermann A 2017 Populations headed south? The Gravettian from a palaeodemographic point of view. Antiquity 91, 573–588. (10.15184/aqy.2017.37)

[53] Schmidt I, Zimmermann A 2019 Population dynamics and socio-spatial organization of the Aurignacian: scalable quantitative demographic data for western and central Europe. PLoS ONE 14, e0211562 (10.1371/journal.pone.0211562) 30759115 PMC6373918

[54] Zimmermann A, Hilpert J, Wendt KP 2009 Estimations of population density for selected periods between the Neolithic and AD 1800. Hum. Biol. 81, 357–380. (10.3378/027.081.0313) 19943751

[55] Binford LR 2001 *Constructing frames of reference: an analytical method for archaeological theory building using hunter-gatherer and environmental data sets*. Berkeley, CA: University of California Press.

[56] Schmidt I et al. 2021 Approaching prehistoric demography: proxies, scales and scope of the Cologne Protocol in European contexts. Phil. Trans. Roc. Soc. B 376, 20190714 (10.1098/rstb.2019.0714) PMC7741091 33250025

[57] Högberg A, Olausson D 2007 Scandinavian flint—an archaeological perspective. Aarhus, Denmark: Aarhus University Press.

[58] Kretschmer I, Maier A, Schmidt I, 2016 Probleme und mögliche Lösungen bei der Schätzung von Bevölkerungsdichten im Paläolithikum. In Alles was zählt (eds Kerig TK, Nowak G). Festschrift für Andreas Zimmermann.

[59] Riede F 2014 The resettlement of Northern Europe. In The Oxford handbook of the archaeology and anthropology of hunter-gatherers (eds Cummings V, Jordan P, Zvelebil M), pp. 556–581. Oxford, UK: Oxford University Press.

[60] Breivik HM 2016 Dynamic relations between humans and environment in the earliest settlement phase of Norway (9500–8000 cal BC), Doctoral theses at NTNU, NTNU, Trondheim, Norway.

[61] Ivanovaitė L, Serwatka K, Hoggard C, Sauer F, Riede F 2019 All these fantastic cultures? Research history and regionalization in the Late Palaeolithic tanged point cultures of Eastern Europe. Eur. J. Archaeol. 23, 162–185. (10.1017/eaa.2019.59)

[62] R Core Team 2020 *R: a Language and environment for statistical computing.* Vienna, Austria: R Foundation for Statistical Computing.

[63] Lundström V, Peters R, Riede F 2021 Demographic estimates from the Palaeolithic–Mesolithic boundary in Scandinavia: comparative benchmarks and novel insights. Phil. Trans. R. Soc. B 376, 20200037 (10.1098/rstb.2020.0037) 33250035 PMC7741095

[64] Preparata F, Shamos M 1985 Computational geometry. New York, NY: Springer.

[65] Burt W 1943 Territoriality and home range concepts as applied to mammals. J. Mammal. 24, 346 (10.2307/1374834)

[66] Grove M, Pearce E, Dunbar R 2012 Fission-fusion and the evolution of hominin social systems. J. Hum. Evol. 62, 191–200. (10.1016/j.jhevol.2011.10.012) 22197359

[67] Crema E 2014 A simulation model of fission–fusion dynamics and long-term settlement change. J. Archaeol. Method Theory 21, 385–404. (10.1007/s10816-013-9185-4)

[68] Fuglestvedt I 2012 The pioneer condition on the Scandinavian Peninsula: the last frontier of a ‘Palaeolithic Way’ in Europe. Norwegian Archaeol. Rev. 45, 1–29. (10.1080/00293652.2012.669998)

[69] Berg-Hansen IM 2017 Continuity and change in Late Glacial and Postglacial social networks; knowledge transmission and blade production methods in Ahrensburgian and Early Mesolithic North West Europe. In The technology of early settlement in Northern Europe transmission of knowledge and culture, vol. 2. (Sheffield 2017) (eds Knutsson K, Knutsson H, Apel J, Glørstad H), pp. 63–98. Sheffield, UK: Equinox Publishing.

[70] Boethius A, Ahlström T 2018 Fish and resilience among Early Holocene foragers of southern Scandinavia: a fusion of stable isotopes and zooarchaeology through Bayesian mixing modelling. J. Archaeol. Sci. 93, 196–210. (10.1016/j.jas.2018.02.018)

[71] Surovell T, Brantingham P 2007 A note on the use of temporal frequency distributions in studies of prehistoric demography. J. Archaeol. Sci. 34, 1868–1877. (10.1016/j.jas.2007.01.003)

[72] Surovell T, Byrd Finley J, Smith G, Brantingham P, Kelly R 2009 Correcting temporal frequency distributions for taphonomic bias. J. Archaeol. Sci. 36, 1715–1724. (10.1016/j.jas.2009.03.029)

[73] Ames KM 2002 Going by boat: the forager-collector continuum at sea. In Beyond foraging and collecting: evolutionary change in hunter-gatherer settlement systems (eds Ben Fitzhugh, Junko Habu), pp. 19–52. New York, NY: Kluwer Academic/Plenum.

[74] Breivik H 2014 Palaeo-oceanographic development and human adaptive strategies in the Pleistocene–Holocene transition: a study from the Norwegian coast. Holocene 24, 1478–1490. (10.1177/0959683614544061)

[75] Gamble C, Davies W, Pettitt P, Hazelwood L, Richards M. 2006 The Late Glacial ancestry of Europeans: combining genetic and archaeological evidence. Documenta Praehistorica 13, 1–10. (10.4312/dp.33.1)

[76] Maier A 2015 The central European Magdalenian: regional diversity and internal variability, vertebrate paleobiology and paleoanthropology series. Dordrecht, The Netherlands: Springer.

[77] Donahue RE, Fischer A 2015 A Late Glacial family at Trollesgave, Denmark. J. Archaeol. Sci. 54, 313–324. (10.1016/j.jas.2014.12.018)

[78] Björck S. 1995 A review of the history of the Baltic Sea, 13.0-8.0 ka BP. Quat. Int. 27, 19–40. (10.1016/1040-6182(94)00057-C)

[79] Brooks AJ, Bradley SL, Edwards RJ, Goodwyn N. 2011 The palaeogeography of Northwest Europe during the last 20,000 years. J. Maps 7, 573–587. (10.4113/jom.2011.1160)

[80] Cohen KM, Westley K, Erkens G, Hijma MP, Weerts HJT 2017 The North Sea. In: Submerged landscapes of the European Continental Shelf (eds Flemming NC, Harff J, Moura D, Burgess A, Bailey GN), pp. 147–186. Oxford, UK: John Wiley & Sons, Ltd.

[81] Edwards RJ, Brooks AJ. 2008 The island of Ireland: drowning the myth of an Irish land-bridge? In: *Mind the gap: postglacial colonisation of Ireland. Special Supplement to The Irish Naturalists journal* (eds JJ Davenport, DP Sleeman, PC Woodman), pp. 19–34. Totnes, UK: NHBS.

[82] Harff J et al 2017 Sea level and climate. In *Submerged landscapes of the European Continental Shelf* (eds NC Flemming, J Harff, D Moura, A Burgess, GN Bailey), pp. 11–49. Oxford, UK: John Wiley & Sons, Ltd.

[83] Lericolais G. 2017 *Late Pleistocene environmental factors defining the Black Sea, and submerged landscapes on the Western Continental Shelf.* In *Submerged landscapes of the European Continental Shelf* (eds NC Flemming, J Harff, D Moura, A Burgess, GN Bailey), pp. 479–495. Oxford, UK: John Wiley & Sons, Ltd.

[84] Moscon G, Corregiari A, Stefani C, Fontana A, Remia A. 2015 Very-high resolution analysis of a transgessive deposit in the Northern Adriactic sea (Italy). Alpine Med. Quat. 28, 121–129.

[85] Påsse T, Andersson L 2005 Shore-level displacement in Fennoscandia calculated from empirical data. GFF 127, 253–268. (10.1080/11035890501274253)

[86] Seguinot J, Ivy-Ochs S, Jouvet G, Huss M, Funk M, Preusser F 2018 Modelling last glacial cycle ice dynamics in the Alps. The Cryosphere 12, 3265–3285. (10.5194/tc-12-3265-2018)

[87] Stroeven AP et al. 2016 Deglaciation of Fennoscandia. Quat. Sci. Rev. 147, 91–121. (10.1016/j.quascirev.2015.09.016)

[88] Subetto D, Zobkov M, Potakhin M, Tarasov A. (2017). https://www.arcgis.com/apps/MapJournal/index.html?appid=47d76ba2004e463d96eba1d8a1825fe1.

[89] Weaver AJ 2003 Meltwater Pulse 1A from Antarctica as a trigger of the Bølling-Allerød Warm Interval. Science 299, 1709–1713. (10.1126/science.1081002) 12637739

[90] Patton H et al. 2017 Deglaciation of the Eurasian ice sheet complex. Quat. Sci. Rev. 169, 148–172. (10.1016/j.quascirev.2017.05.019)

[91] Pasanen A, Lunkka JP, Putkinen N 2010 Reconstruction of the White Sea Basin during the late Younger Dryas. Boreas 39, 273–285. (10.1111/j.1502-3885.2009.00128.x)

[92] Larsson L 1996 The colonization of southern Sweden during the deglaciation of Scandinavia and its relationship with neighbouring areas. Acta Archaeologica Lundensia Series in 8°, 24 (Stockholm 1996) 141–155.

[93] Mortensen M, Henriksen P, Bennike O 2014 Living on the good soil: relationships between soils, vegetation and human settlement during the late Allerød period in Denmark. Veg. Hist. Archaeobot. 23, 195–205. (10.1007/s00334-014-0433-7)

[94] Surovell T 2000 Early Paleoindian women, children, mobility, and fertility. Am. Antiq. 65, 493–508. (10.2307/2694532) 17086673

[95] White A 2017 A model-based analysis of the minimum size of demographically-viable hunter-gatherer populations. J. Artif. Soc. Soc. Simul. 20 (10.18564/jasss.3393)[AQ4]

[96] Boone JL 2002 Subsistence strategies and early human population history: an evolutionary ecological perspective. World Archaeol. 34, 6–25. (10.1080/00438240220134232) 16475305

[97] Laughlin CD, Brady IA 1978 Extinction and survival in human populations. New York, NY: Columbia University Press.

[98] Mandryk CAS 1993 Hunter-gatherer social costs and the nonviability of submarginal environments. J. Anthropol. Res. 49, 39–71. (10.1086/jar.49.1.3630629)

[99] Minc LD, Smith KP 1989 The spirit of survival. In Bad year economics: cultural responses to risk and uncertainty (eds Halstead P, O'Shea J), pp. 8–39. Cambridge, UK: Cambridge University Press.

[100] Paine RR 1997 Integrating archaeological demography: multidisciplinary approaches to prehistoric population. Carbondale, IL: Center for Archaeological Investigations, Southern Illinois University at Carbondale.

[101] Fagan W, Holmes E 2005 Quantifying the extinction vortex. Ecol. Lett. 9, 51–60. (10.1111/j.1461-0248.2005.00845.x) 16958868

[102] Premo L, Kuhn S 2010 Modeling effects of local extinctions on culture change and diversity in the Paleolithic. PLoS ONE 5, e15582 (10.1371/journal.pone.0015582) 21179418 PMC3003693

[103] Riede F, Edinborough K 2012 Bayesian radiocarbon models for the cultural transition during the Allerød in southern Scandinavia. J. Archaeol. Sci. 39, 744–756. (10.1016/j.jas.2011.11.008)

[104] Bjerck H 2009 Colonizing seascapes: comparative perspectives on the development of maritime relations in Scandinavia and Patagonia. Arctic Anthropol. 46, 118–131. (10.1353/arc.0.0019)

[105] Bang-Andersen S 2003 Southwest Norway at the Pleistocene/Holocene transition: landscape development, colonization, site types, settlement patterns. Norwegian Archaeol. Rev. 36, 5–25. (10.1080/00293650307293)

[106] Glørstad H 2014 Deglaciation, sea-level changes and the Holocene colonisation of Norway. In Geology and archaeology: submerged landscapes of the continental shelf, Geological Society, vol 411. Special publications, London (eds Harff J, Bailey G, Lüth F), pp. 9–25. London, UK: The Geological Society.

[107] Kindgren H 1995 Hensbacka-Horgen-Hornborgarsjön: early Mesolithic coastal and inland settlement in Western Sweden. In Man and sea in the Mesolithic. Oxbow monograph, 53 (ed Fischer A), pp. 171–184. Oxford, UK: Oxbow Books.

[108] Kindgren H, 1996 Reindeer or seals? Some Late Palaeolithic sites in Central Bohuslän. In The earliest settlement of Scandinavia and its relationship with neighbouring areas (ed. Larsson L), pp. 193–203. Acta Archaeologica Lundensia, 24 Stockholm, Sweden: Almquist and Wiksell International.

[109] Schmitt L, Larsson S, Burdukiewicz J, Ziker J, Svedhage K, Zamon J, Steffen H 2009 Chronological insights, cultural change, and resource exploitation on the west coast of Sweden during the late Palaeolithic/early Mesolithic transition. Oxford J. Archaeol. 28, 1–27. (10.1111/j.1468-0092.2008.00317.x)

[110] Jørgensen EK 2020 The palaeodemographic and environmental dynamics of prehistoric Arctic Norway: an overview of human-climate covariation. Quat. Int. 549, 36–51. (10.1016/j.quaint.2018.05.014)

[111] Solheim S, Persson P 2018 Early and mid-Holocene coastal settlement and demography in southeastern Norway: comparing distribution of radiocarbon dates and shoreline-dated sites, 8500–2000 cal. BCE. J. Archaeol. Sci. Rep. 19, 334–343. (10.1016/j.jasrep.2018.03.007)

[112] Smith CM 2014 Estimation of a genetically viable population for multigenerational interstellar voyaging: review and data for project Hyperion. Acta Astronaut. 97, 16–29. (10.1016/j.actaastro.2013.12.013)

[113] Gjerde JM.S., Hole JT, 2013 Tønsnes Havn, Tromsø kommune, Troms. Rapport frå dei arkeologiske undersøkingane 2011 og 2012. UiT Norges arktiske universitet See https://hdl.handle.net/10037/5837

[114] Breivik HM, Fossum G, Solheim S 2018 Exploring human responses to climatic fluctuations and environmental diversity: two stories from Mesolithic Norway. Quat. Int. 465, 258–275. (10.1016/j.quaint.2016.12.019)

[115] Bergsvik KA 2001 Sedentary and mobile hunter-fishers in Stone Age western Norway. Arctic Anthropol. 38, 2–26.

[116] Bang-Andersen S 2012 Colonizing contrasting landscapes. The pioneer coast settlement and inland utilization in southern Norway 10 000–9500 years before present. Oxford J. Archaeol. 31, 103–120. (10.1111/j.1468-0092.2012.00381.x)

[117] French JC 2015 The demography of the Upper Palaeolithic hunter–gatherers of Southwestern France: a multi-proxy approach using archaeological data. J. Anthropol. Archaeol. 39, 193–209. (10.1016/j.jaa.2015.04.005)

[118] Olsen AB, Alsaker S 1984 Greenstone and diabase utilization in the stone age of western Norway: technological and socio-cultural aspects of axe and adze production and distribution. Nor. Archaeol. Rev. 17, 71–103. (10.1080/00293652.1984.9965401)

[119] Weninger B et al. 2008 The catastrophic final flooding of Doggerland by the Storegga Slide tsunami. Documenta Praehistorica 35, 1–24. (10.4312/dp.35.1)

[120] Rogers AR 1992 Resources and population dynamics. In Evolutionary ecology and human behavior (eds Smith EA, Winterhalder B), pp. 375–402. New York, NY: Aldine de Gruyter.

[121] Sterelny K 2020 Demography and cultural complexity. Synthese (10.1007/s11229-020-02587-2)

