# Supplementary material for: Demographic estimates from the Palaeolithic–Mesolithic boundary in Scandinavia: comparative benchmarks and novel insights
Source: Philos Trans R Soc Lond B Biol Sci. 2020 Nov 30;376(1816):20200037. doi: 10.1098/rstb.2020.0037 (PMC7741095; doi:10.1098/rstb.2020.0037)
Supplement: Supplementary information and figures [file rstb20200037supp1.docx]

Supplementary Information

**Demographic estimates from the Palaeolithic-Mesolithic boundary in Scandinavia: Comparative benchmarks and novel insights**

Victor Lundström^1^ Robin Peters^2^ Felix Riede^3,4^

**Table of Contents**

[1. Data and study area 2](#_Toc48721578)

[2. Preparation before Ordinary Kriging 3](#_Toc48721579)

[2.1. Voronoi diagram and density measurements. 3](#_Toc48721580)

[2.1.1. Semivariograms 4](#_Toc48721581)

[2.1.2. Kriging rasters 5](#_Toc48721582)

[2.1.3. ODI selection 6](#_Toc48721583)

[3. Deriving demographic parameters 7](#_Toc48721584)

[3.1. Ethnographic reference groups 7](#_Toc48721585)

[3.2. Adjusting the protocol 8](#_Toc48721586)

[4. Results 10](#_Toc48721587)

[4.1. Late Glacial and Early Holocene estimates: GROUP1 hunter-gatherers 10](#_Toc48721588)

[5. Comparative estimates: GROUP1 & GROUP2 11](#_Toc48721589)

[5.1. The Late Glacial Final Palaeolithic 11](#_Toc48721590)

[5.2. Early Holocene Norway 12](#_Toc48721591)

[6. Distribution maps 13](#_Toc48721592)

[7. Literature 14](#_Toc48721593)

1. University Museum, University of Bergen, Villaveien 1A, 5020 Bergen, Norway; victor.lundstrom@uib.no

2. LVR-State Service for Archaeological Heritage, Endenicher Str. 133, 53115 Bonn, Germany; [mail@robinpeters.net](mailto:mail@robinpeters.net)

3. Department of Archaeology and Heritage Studies, Aarhus University, Moesgård Allé 20, 8270 Højbjerg, Denmark; [f.riede@cas.au.dk](mailto:f.riede@cas.au.dk)

4. BIOCHANGE - Centre for Biodiversity Dynamics in a Changing World, Aarhus University, Ny Munkegade 116 DK-8000 Aarhus C, Denmark

# 1. Data and study area

Original data and results for the study, can be found here: <https://github.com/VicluUiB/Demographic-estimates-from-the-Palaeolithic-Mesolithic-boundary_PhilTransB2020>. The R-markdown for performing the geostatistical component of the protocol is accessed at: <https://github.com/C-C-A-A/CologneProtocol-R>. Both data sets cover the modern-day administrational boundaries (Fig.1) of Denmark and the southernmost province of Scania in Sweden (solid line) as well as the entire Norwegian coast (dashed line).


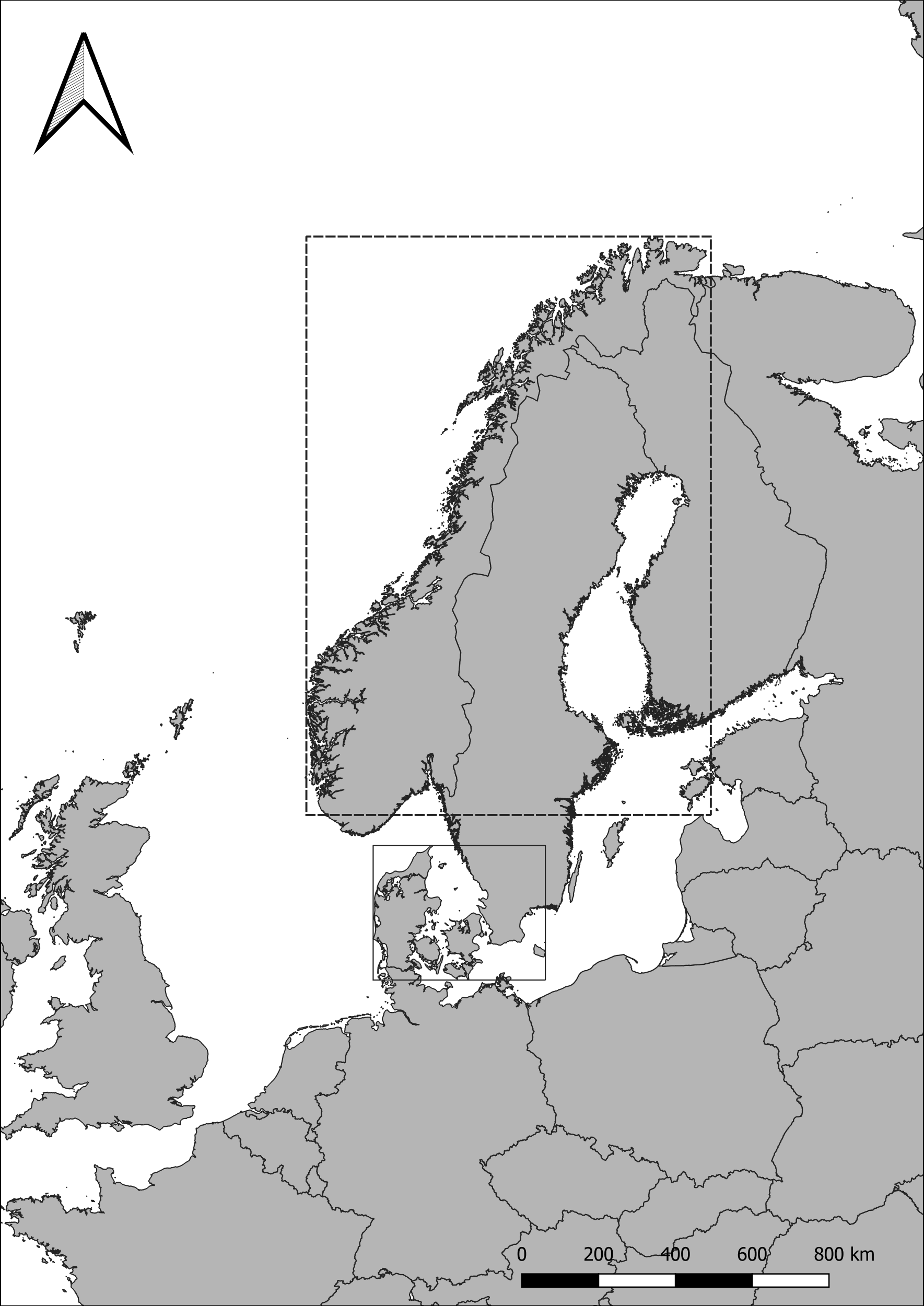


Figure 1. Modern administrational boundaries were obtained from [www.gadm.org](http://www.gadm.org). Projection: UTM32N; EPSG: 25832.

# 2. Preparation before Ordinary Kriging

## 2.1. Voronoi diagram and density measurements.

| *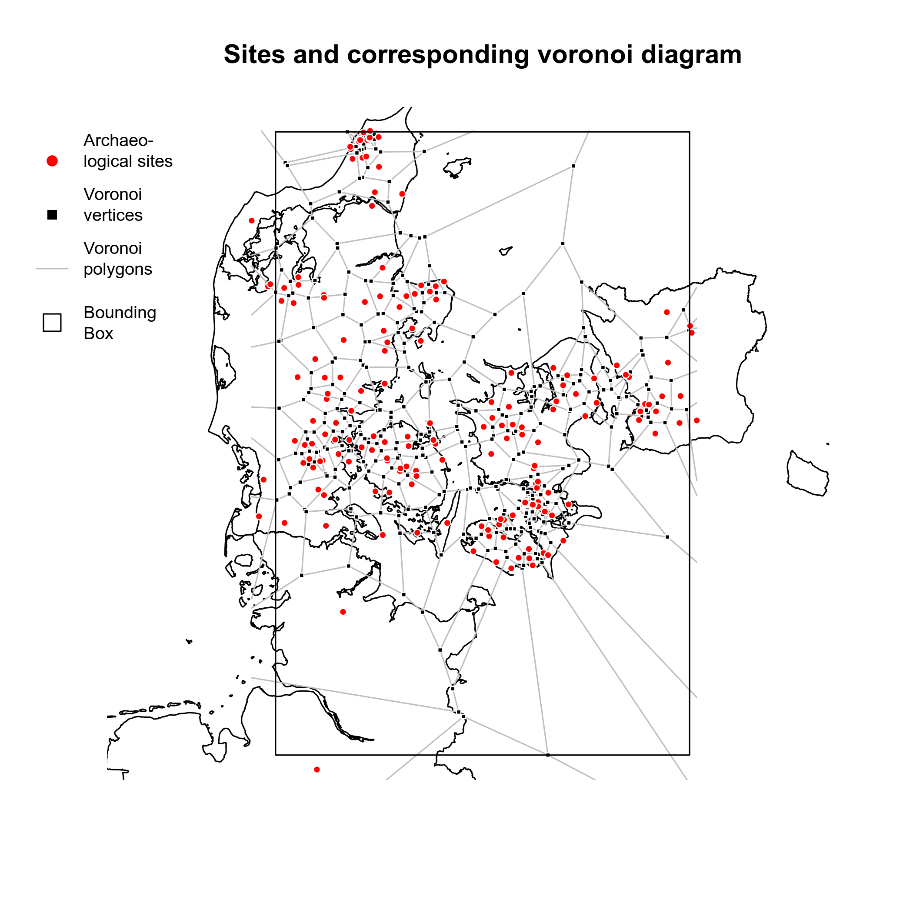* | 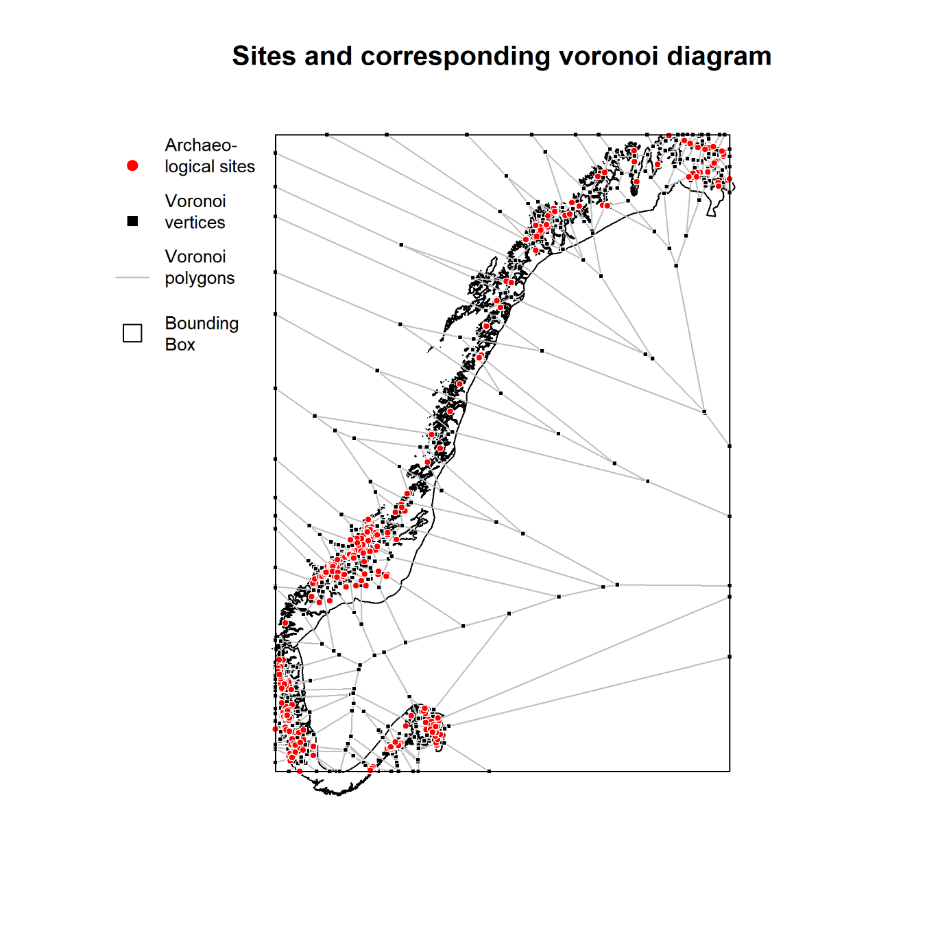 |
| --- | --- |

Figure 2. We start by plotting our site locations, followed by producing a Voronoi diagram (grey lines) as well as extracting the largest empty circle measures (black vertices) for the geostatistical interpolation of site densities. These are plotted within the modern-day landmasses of northern Germany, Denmark, Scania (left panel) and Norway (right panel) and forms the so-called “total area of calculation” (TAC) and its area in km^2^ constitutes the input for which metapopulation densities are calculated. Projection: UTM32N; EPSG: 25832

### 2.1.1. Semivariograms

| *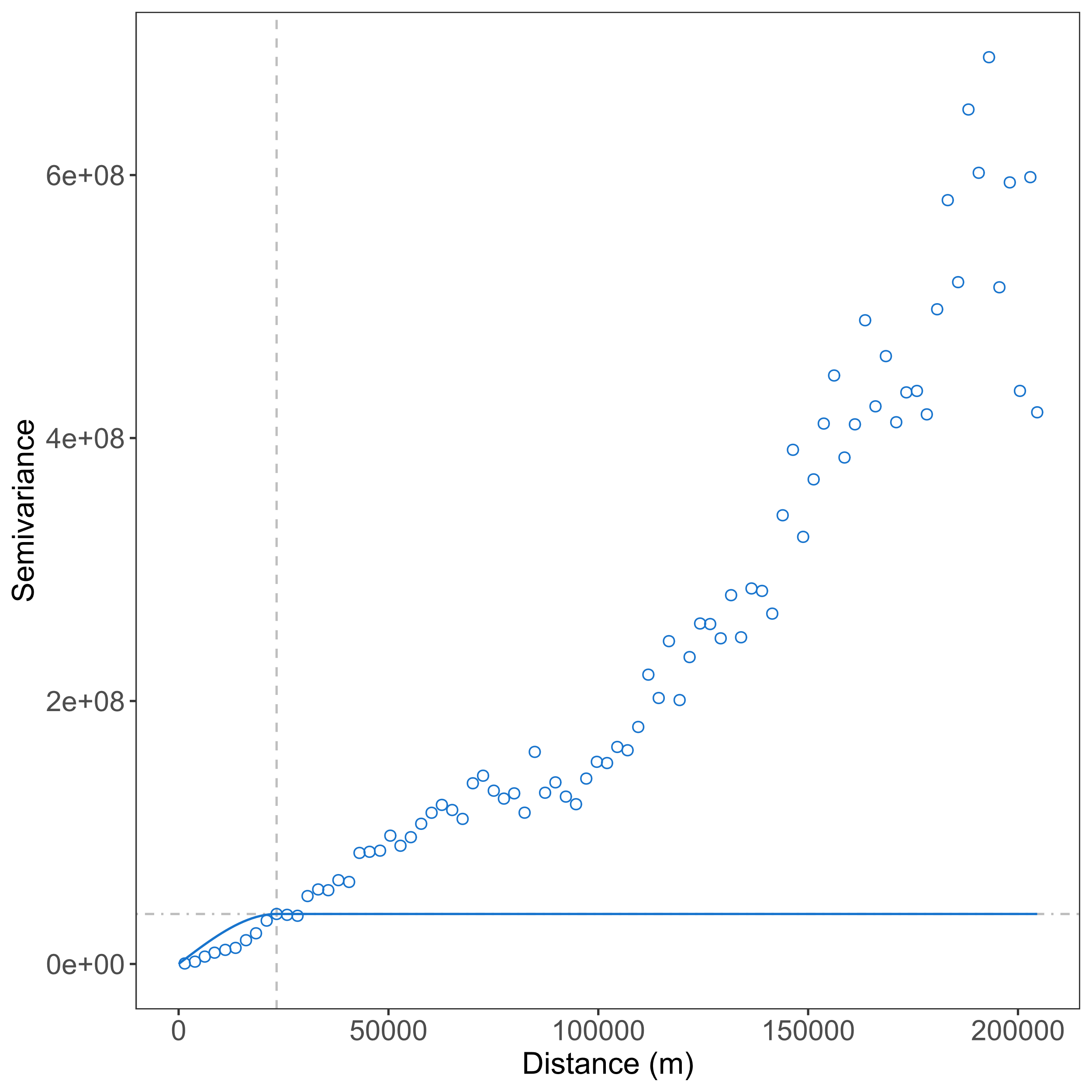* | *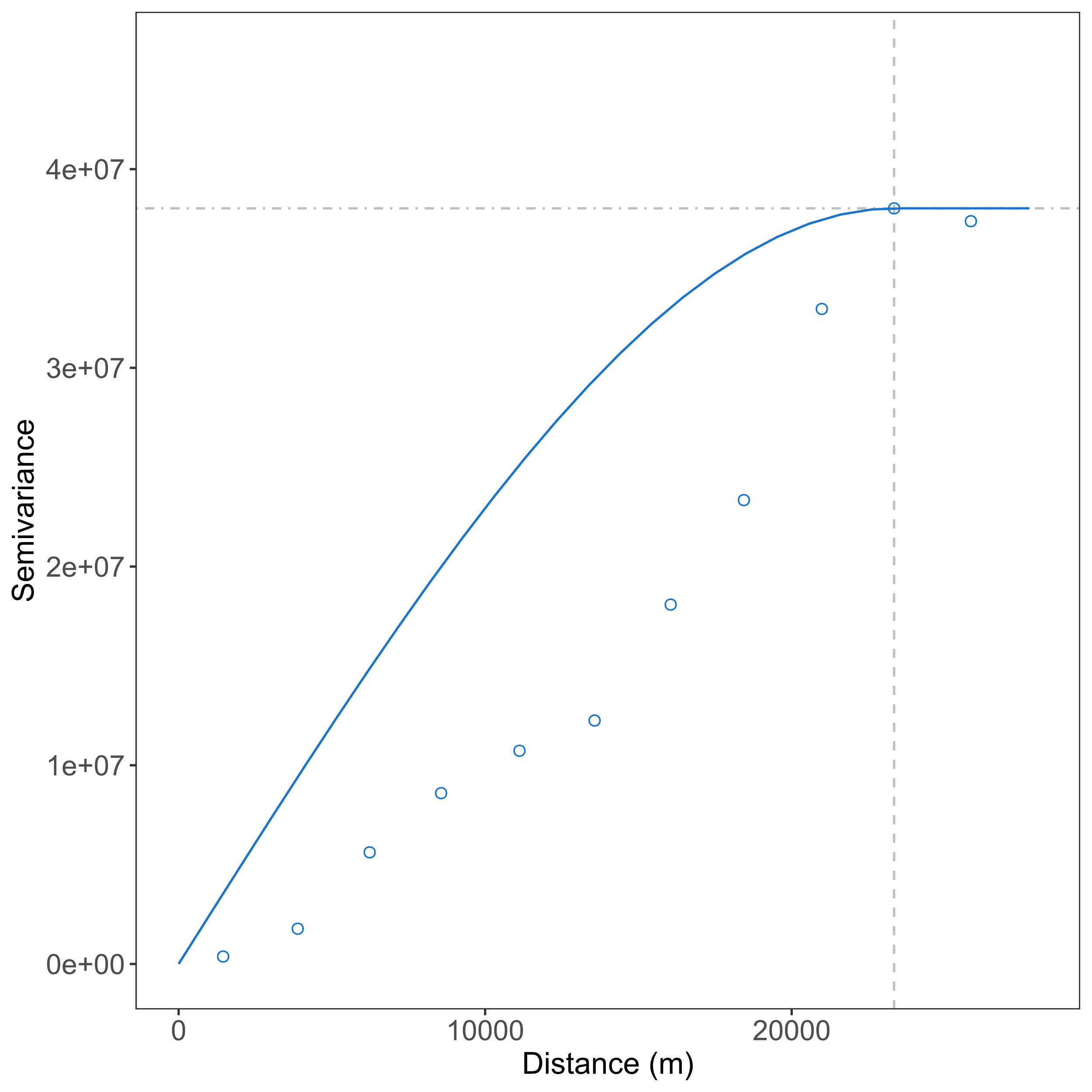* |
| --- | --- |
| 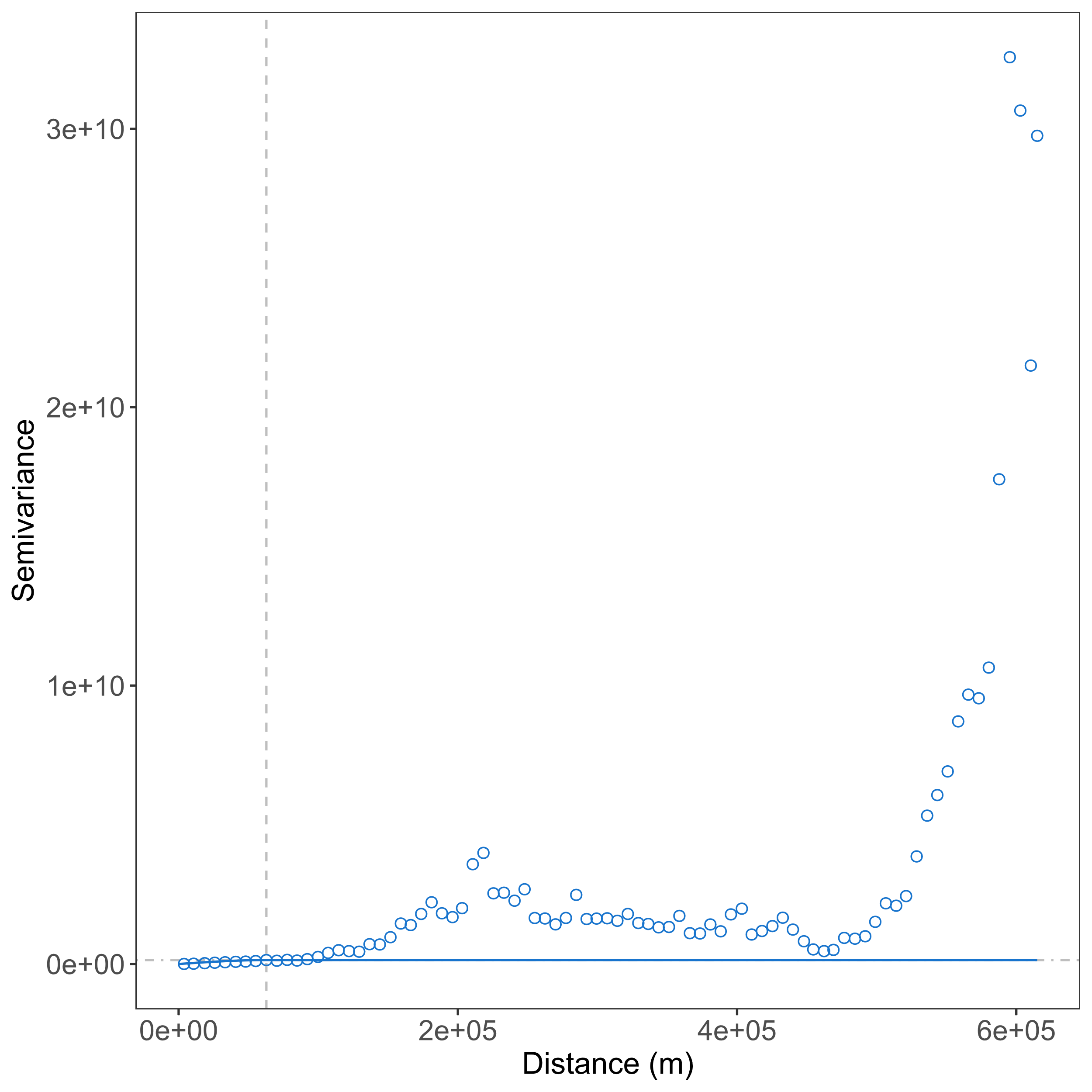 | 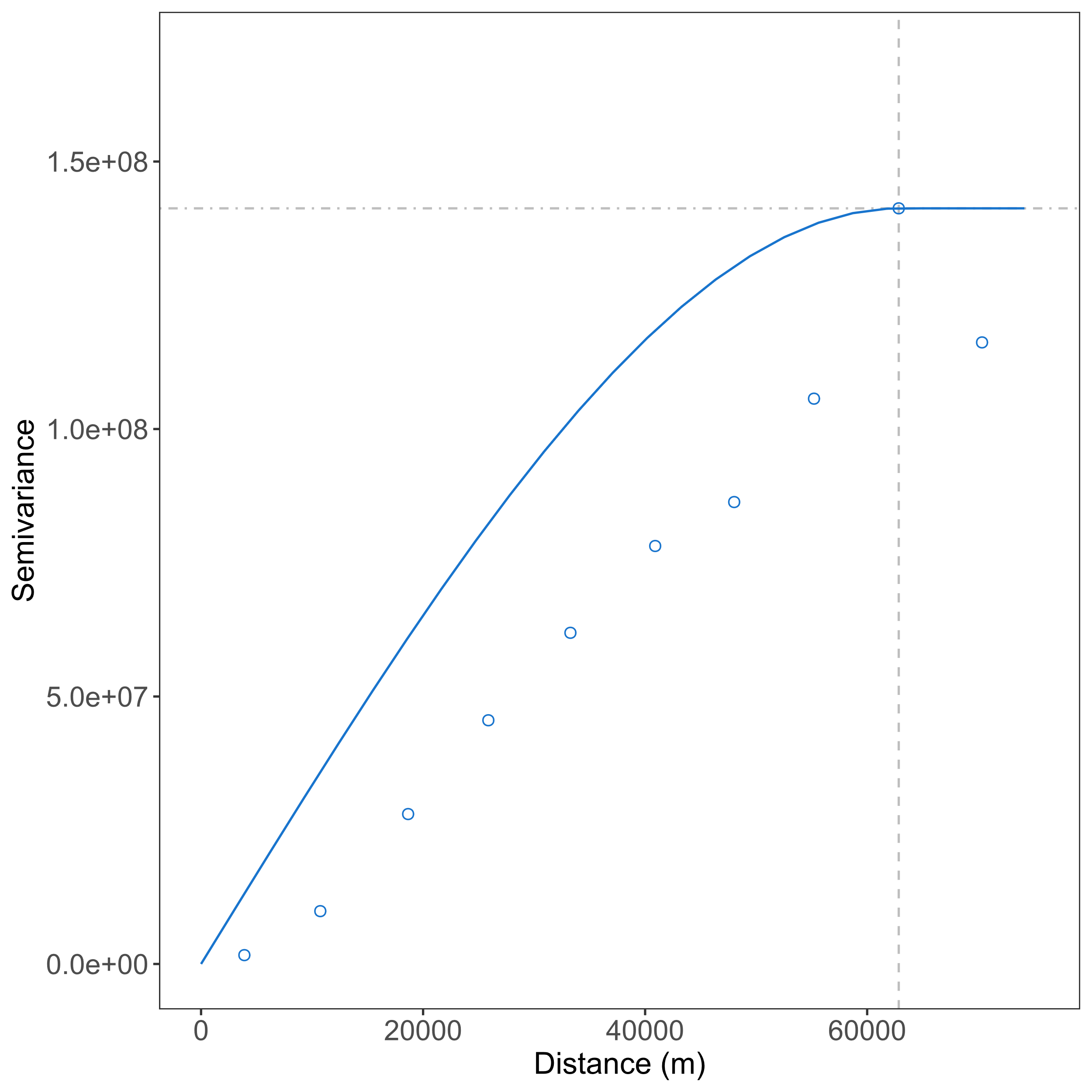 |

Figure 3. We then produce semivariograms for southern Scandinavia (upper panels) and Norway (bottom panels) using a ‘spherical’ model. At a certain distance, our spherical model hits a plateau of no autocorrelation, as indicate by an enlargement of the plots (right panels), meaning that predictions beyond these distances will be highly inaccurate. This distance, called the ‘range’, intersects with the amount of semivariance at which the curve levels off, also known as the ‘sill’ (Isaaks and Srivastava, 1990).

### 2.1.2. Kriging rasters

| 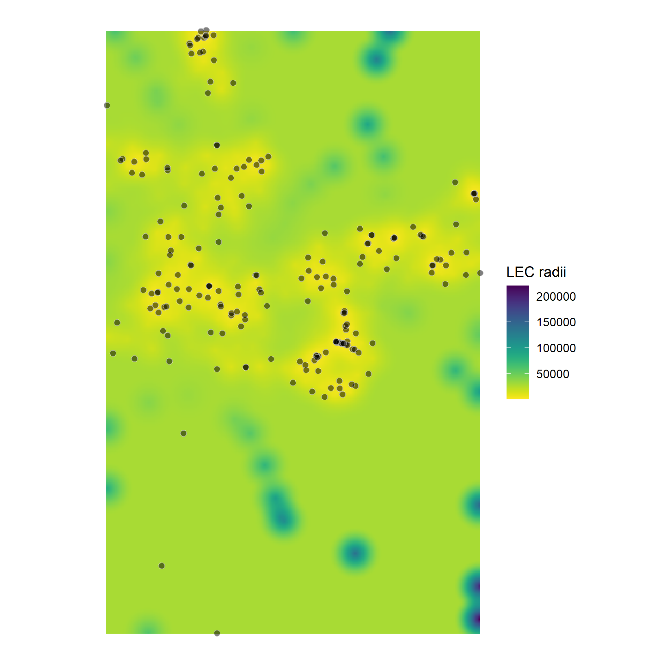 | 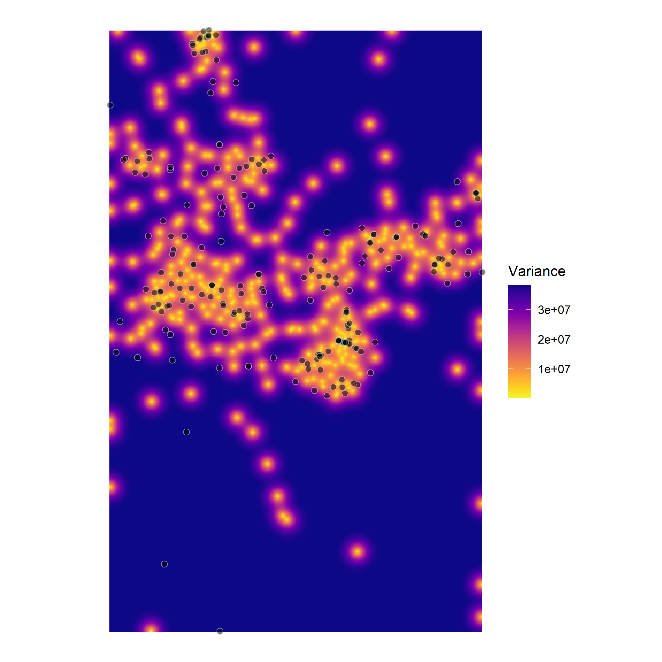 |
| --- | --- |
| 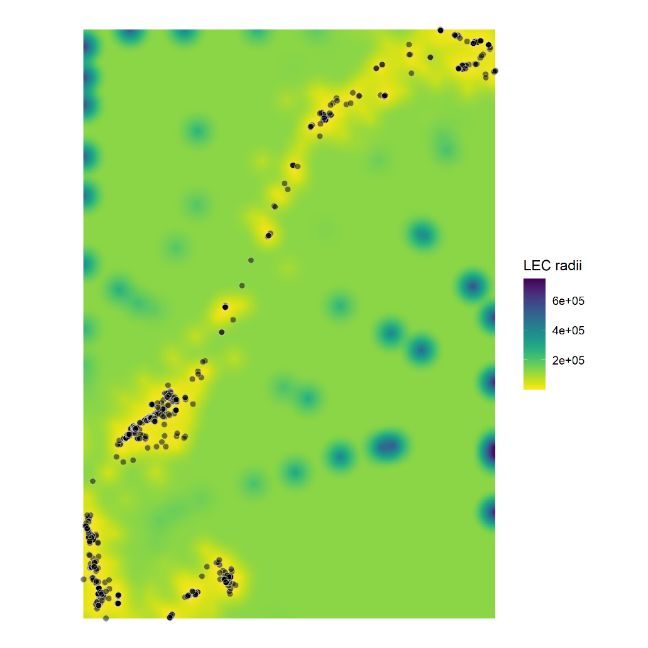 | 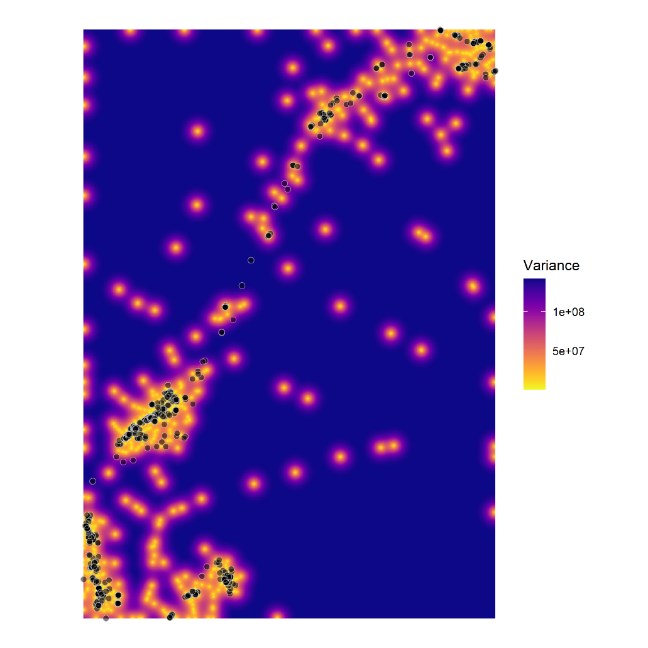 |

Figure 4. Four resulting rasters from the geostatistical interpolation. Sites from southern Scandinavia (upper panels) and Norway (lower panels) are superimposed on all rasters for spatial reference, and the colour palette that ranges from yellow to blue (left panels) indicates the size of the LEC-radii, and therefore settlement density, whereas the palette ranging from yellow to purple (right panels) indicates the amount of kriging variance, or in other words, the degree of predictive uncertainty. Projection: UTM32N; EPSG: 25832.

### 2.1.3. ODI selection

| 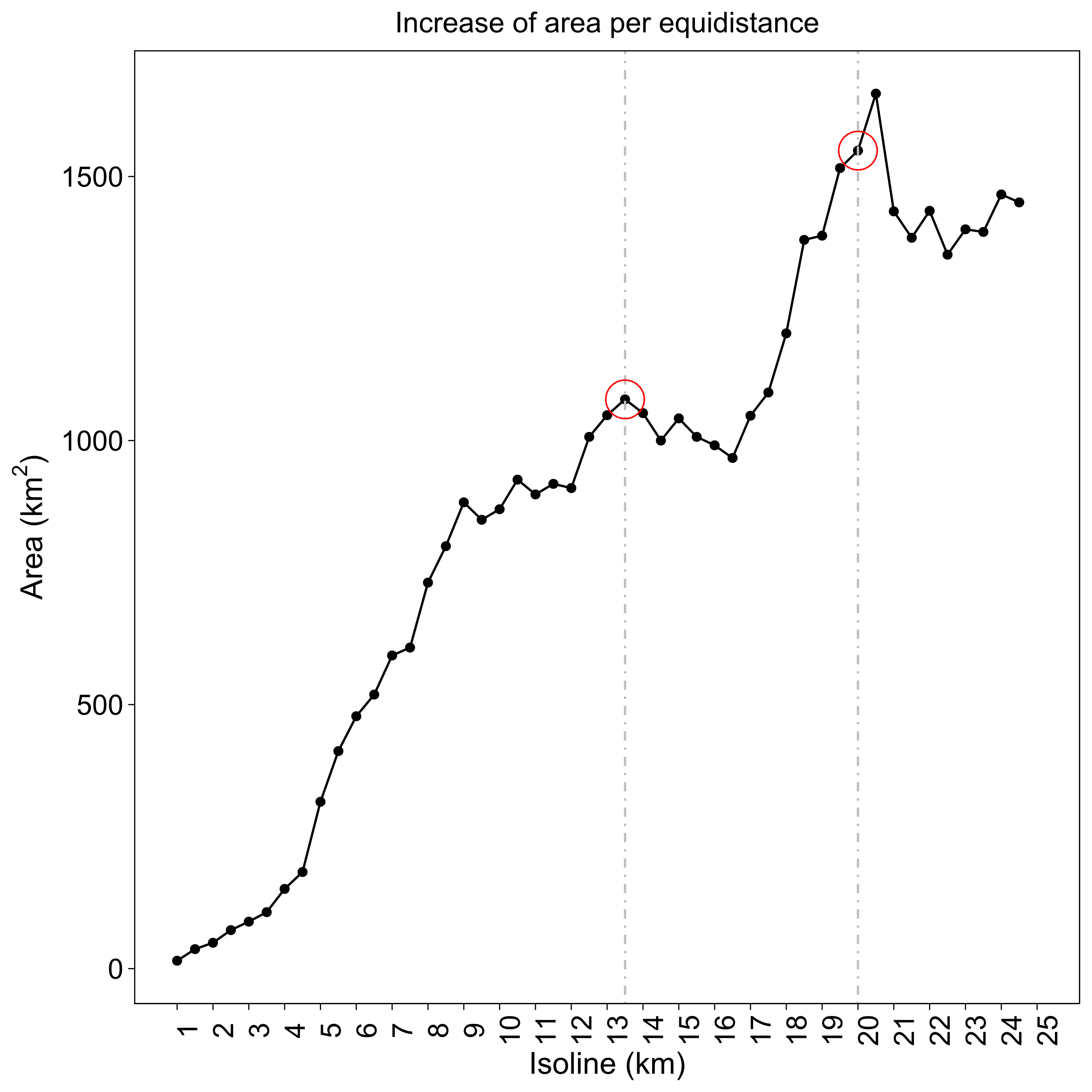 | 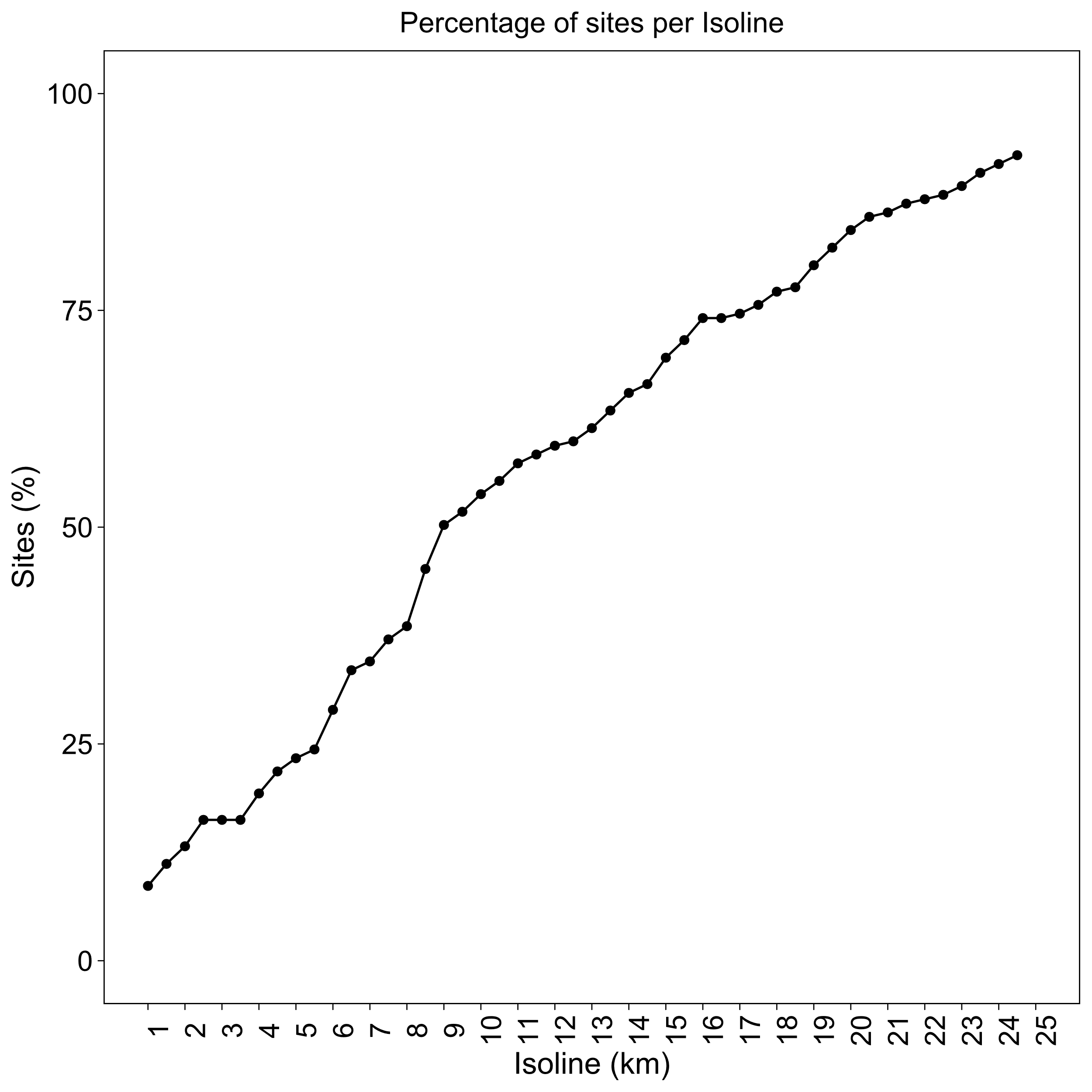 |
| --- | --- |
| 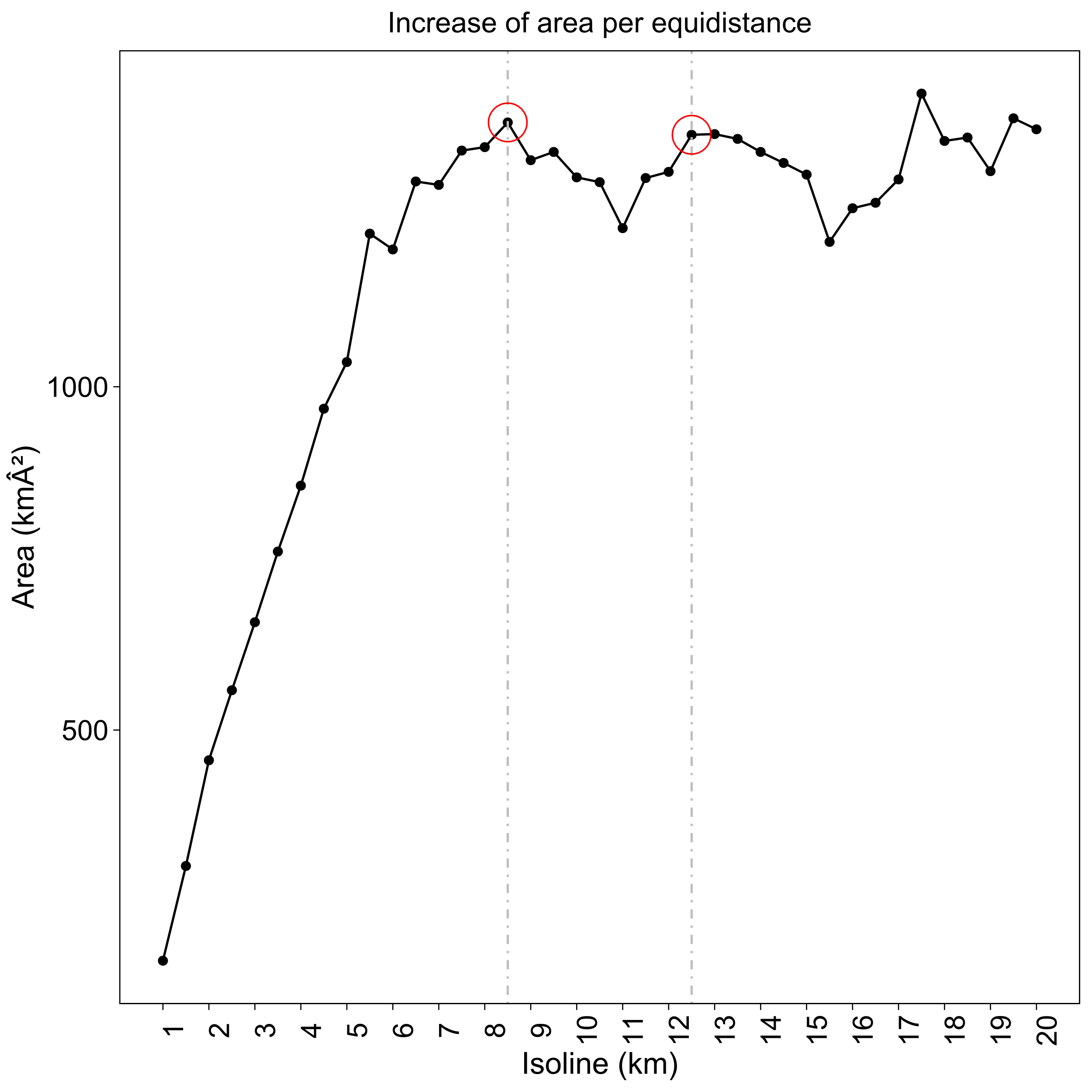 | 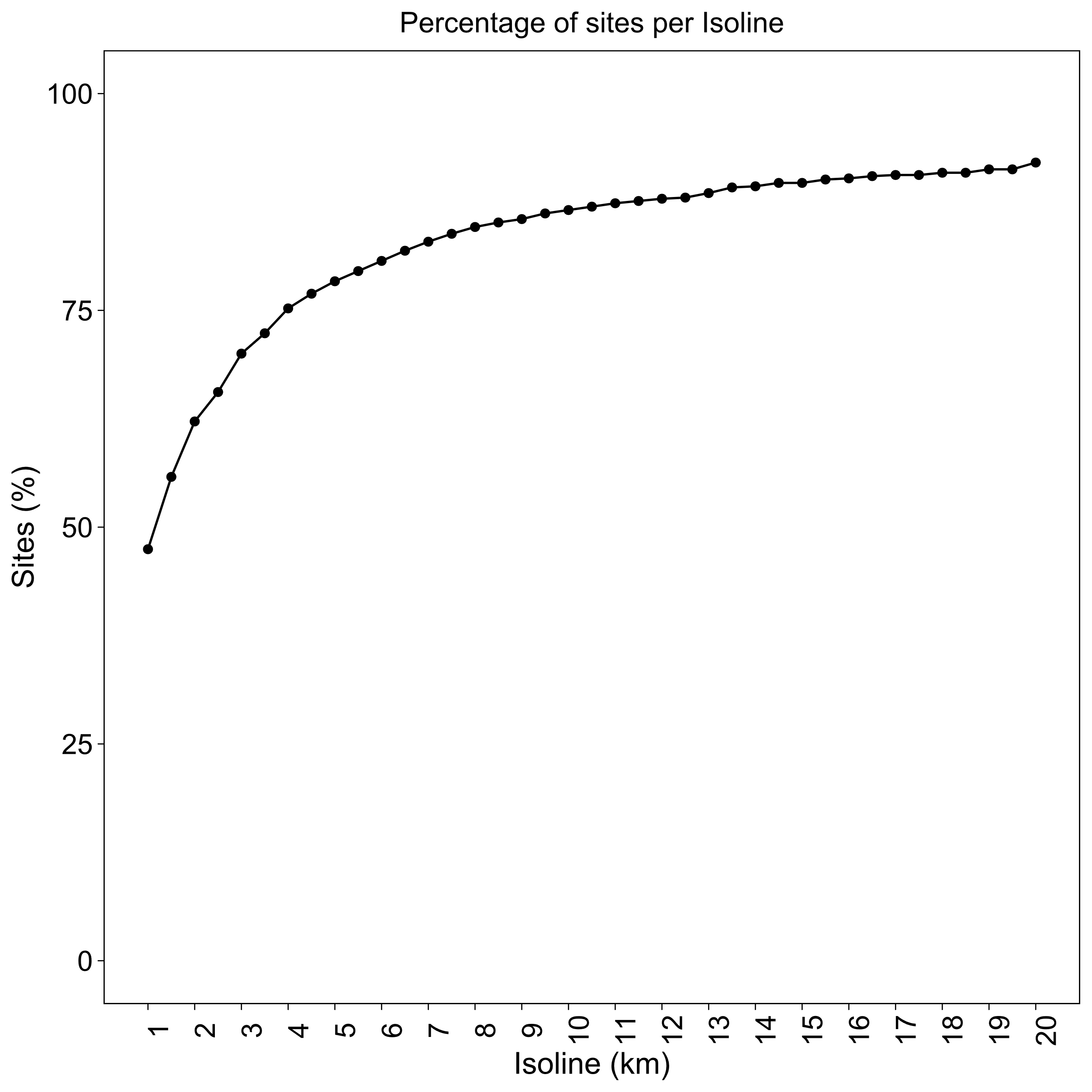 |

Figure 5. The ODI’s for southern Scandinavia (upper panels) and Norway (lower panels) are selected using three heuristic criteria. The ODI for core areas is identified by (1) the first and most significant peak in areal growth per equidistance of the Largest Empty Circle (LEC) radii (left panels) and (2) should encircle approximately 70-90% of all site locations (right panels). The ODI for home ranges should represent a second peak in areal growth and shall, together with the ODI for core areas, (3) remain within an accepted range of kriging variance, which we have defined as anywhere between the lowest amount of kriging variance and the sill. For southern Scandinavia, ODI’s for core areas and home ranges were selected at a 13.5 and 20 km equidistance. The second peak for home ranges actually occurs at 20.5km equidistance, however, this turned our study area into one single home range, preventing us to operate with a median estimate. For early Holocene Norway, we opted for an 8.5 km and 12.5 km ODI for core areas and home ranges. 8.5km constitutes a clear peak in growth, whereas 12.5km represents a plateau in growth. There is also a third peak at 17.5km, however, growth seems to fluctuate somewhat in these parts, perhaps as a result of increased kriging variance. To minimize uncertainties in our estimates, we therefore select the ODI at 12.5km for home ranges.

# 3. Deriving demographic parameters

## 3.1. Ethnographic reference groups

**Table 1.** Due to a highly fragmented archaeological record in both case studies, we have to rely on selection of ethnographic reference data from previous research (see Kretschmer for an outline of their selection criteria, but also Lundström and Riede, 2019; Schmidt and Zimmermann, 2019). However, in order to produce comparative estimates for the early Holocene, in which populations might have relied more fully on an aquatic economy, we have selected nine ethnographic reference groups on the basis of stable isotope data from the site Huseby Klev (Boethius and Alhström, 2018) that, however, post-date our time-slice to the end of the preboreal. As this makes our application of these groups highly problematic, we will focus our modelling mainly on the more terrestrially oriented groups mentioned earlier, using more aquatic oriented groups only as comparanda (denoted in italics). By slightly relaxing our selection criteria to groups that are mobile and who obtain 65-75% in aquatic subsistence, 5-35% terrestrial and 0-20% plant gathering we end up with nine groups, where six inhabit the northern hemisphere, and three inhabit the southern hempisphere. Once again, these are mainly used as comparanda, our main focus lies on the more terrestrially oriented groups.

| **Group#** | **Name** | **Latitude** | **Longitude** | **GROUP1** | **GROUP2** |
| --- | --- | --- | --- | --- | --- |
| 335 | Round Lake Ojibwa | 52.71 | 90.62 | 7 | 50 |
| 377 | Nunamiut Inuit | 68.18 | 151.71 | 8.9 | 31 |
| 358 | Satudene | 65.75 | 122.89 | 12 | 29 |
| 352 | Hare | 67.47 | 125 | 13 | 26 |
| 345 | Slave | 61.15 | 119.5 | 13 | 39 |
| 338 | Mistassini Cree | 51.75 | 72.66 | 15 | 37 |
| 332 | North Saulteaux | 52.28 | 96.78 | 15 | 45 |
| 350 | Mountain | 63.68 | 125.37 | 15 | 60 |
| 346 | Kaska | 59.73 | 125 | 16 | 58 |
| 375 | Noatak Inuit | 68.09 | 160 | 17.5 | 30 |
| 343 | Sekani | 56.14 | 120 | 18 | 40 |
| 54 | Ona | 53.9 | 68.62 | 20 | 45 |
| 331 | Rainy River Ojibwa | 45.21 | 85.1 | 21 | 65 |
| 365 | Naskapi | 56.19 | 68.92 | 23 | 39 |
| 355 | Chippewyan | 59.47 | 106.19 | 23 | 75 |
| 347 | Tahltan | 57.66 | 127.85 | n/a | 71 |
| **Group#** | **Name** | **Lat.** | **Long.** | **GROUP1** | **GROUP2** |
| *55* | *Yahgan* | *55.02* | *68.98* | *13* | *24* |
| *383* | *Aivilingmiut Inuit* | *65.16* | *88.12* | *13* | *26* |
| *372* | *Labrador Inuit* | *57.83* | *62.32* | *11* | *35* |
| *390* | *Inughuit (North Greenland)* | *77.53* | *69.50* | *11.5* | *35* |
| *382* | *Utkuhikhalingmiut* | *66.99* | *95.18* | *18* | *35* |
| *387* | *Netsilik Inuit* | *71.46* | *94.93* | *22* | *85* |
| *381* | *Copper Inuit* | *68.58* | *106.61* | *18* | *105* |
| *53* | *Alacaluf* | *49.55* | *74.52* | *13.4* | *n/a* |
| *52* | *Chono* | *45.00* | *73.83* | *n/a* | *n/a* |

## 3.2. Adjusting the protocol

When we model the number of GROUP1 social units, we do so by dividing the median km^2^ of the so-called ‘home range’ with the km^2^ of each core area. The maximum, 75^th^ percentile, median, 25^th^ percentile, and the minimum estimate on group size from the ethnographic record, is then multiplied by the number of groups (Ng). This results in the total number of people (Np) which can then be generated into population densities within core areas (Dca), home ranges (Dhr) and across the much wider region for the metapopulation (Dmp). See Table 2 for a summary. Whenever we make comparisons with GROUP2 social units, the number of groups is instead derived by dividing the km^2^ of each core area, with the median km^2^ of home ranges.
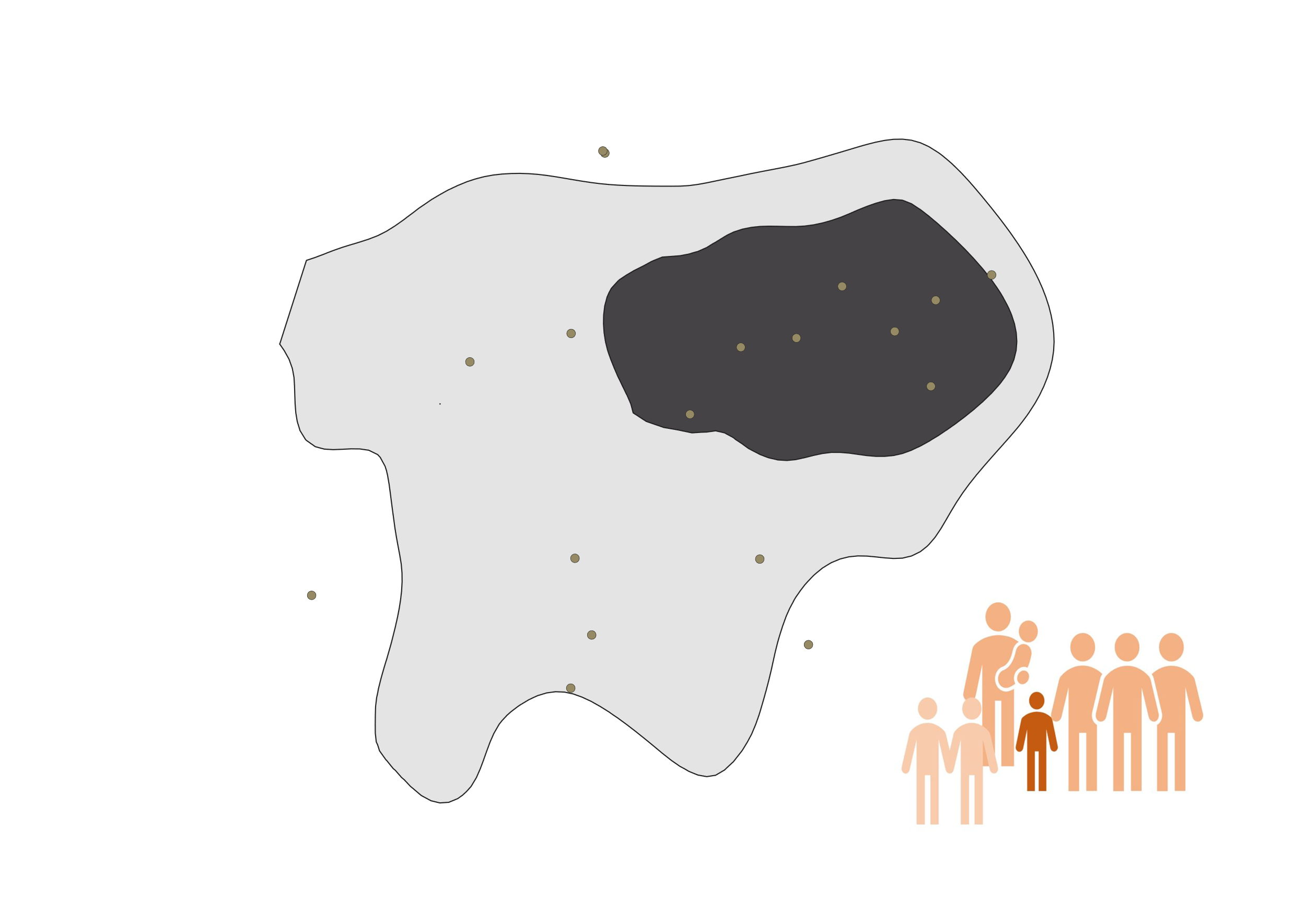
Figure 6*.* Demographic parameters are generated using the fundamental estimate on the number of groups present within each core area. The number of groups (Ng), is obtained by dividing the median km^2^ of home ranges (light grey) with the km^2^ of core areas (dark grey). Colour palette for the figure on human group size was adapted from Riede et al (2019, p.3).

**Table 2** Summary of demographic parameters obtained, and the formulas or mode for calculating them.

| **Protocol outputs** | **Abbreviations** | **Mode of calculation** |
| --- | --- | --- |
| Core Area in km^2^ | Aca | Ordinary kriging & 1^st^ peak ODI |
| Median home Range in km^2^ | Mhr | Ordinary kriging & 2^nd^ peak ODI |
| Number of groups | Ng | Ahr / Aca |
| Group size | Gs | Max, 75^th^, median, 25^th^ and min |
| Number of people | Np | Max, 75^th^, median, 25^th^ and min * Ng |
| Density within core areas | Dca | Np / Aca |
| Density within home ranges | Dhr | Np / Ahr |
| Metapopulation density | Dmp | Np / Atac |
| Total area of calculation in Km^2^ | Atac | Polygons of modern national borders |

# 4. Results

## 4.1. Late Glacial and Early Holocene estimates: GROUP1 hunter-gatherers

**Table 3** Results for southern Scandinavia and Norway (italics). Mhr (median home range in km^2^), Tca (total km^2^ of core areas per region), Ng (number of GROUP1 social units), R (range), Gs (size of GROUP1 social units), Np (total number of people), Dca (population density within core areas), Dhr (population density within home ranges), Atac (km^2^ of total area of calculation), Dmp (metapopulation density).

|  | **Region** | **Mhr** | **Tca** | **Ng** | **R** | **Gs** | **Np** | **Dca** | **Dhr** | **Atac** | **Dmp** |
| --- | --- | --- | --- | --- | --- | --- | --- | --- | --- | --- | --- |
|  |  |  |  |  | Max | 23 | 368 | 0.03 | 0.16 |  | 0.003 |
|  |  |  |  |  | Q3 | 18 | 286 | 0.02 | 0.12 |  | 0.002 |
|  | DK | 2369 | 12,244 | 16 | Median | 15 | 240 | 0.02 | 0.10 | 54,496 | 0.002 |
|  |  |  |  |  | Q1 | 13 | 208 | 0.02 | 0.09 |  | 0.002 |
|  |  |  |  |  | Min | 7 | 112 | 0.01 | 0.05 |  | 0.001 |
|  |  |  |  |  | Max | 23 | 299 | 0.25 | 0.12 |  | 0.002 |
|  |  |  |  |  | Q3 | 18 | 232 | 0.19 | 0.10 |  | 0.002 |
|  | S. Swe | 2369 | 1178 | 13 | Median | 15 | 195 | 0.16 | 0.08 | 54,496 | 0.002 |
|  |  |  |  |  | Q1 | 13 | 169 | 0.14 | 0.07 |  | 0.001 |
|  |  |  |  |  | Min | 7 | 91 | 0.08 | 0.04 |  | 0.001 |
|  |  |  |  |  | Max | 23 | 667 | 0.05 | 0.28 |  | 0.006 |
|  |  |  |  |  | Q3 | 18 | 518 | 0.04 | 0.22 |  | 0.004 |
| Total | S. Scand | 2369 | 13,422 | 29 | Median | 15 | 435 | 0.03 | 0.18 | 54,496 | 0.004 |
|  |  |  |  |  | Q1 | 13 | 377 | 0.03 | 0.16 |  | 0.003 |
|  |  |  |  |  | Min | 7 | 203 | 0.02 | 0.09 |  | 0.002 |
|  | **Region** | **Ahr** | **Tca** | **Ng** | **R** | **Gs** | **Np** | **Dca** | **Dhr** | **Atac** | **Dmp** |
|  |  |  |  |  | *Max* | *23* | *153* | *0.07* | *0.15* |  | *0.001* |
|  |  |  |  |  | *Q3* | *19* | *126* | *0.06* | *0.13* |  | *0.001* |
|  | *SE. Nor* | *990* | *2261* | *7* | *Median* | *15* | *100* | *0.04* | *0.10* | *146,624* | *0.001* |
|  |  |  |  |  | *Q1* | *13* | *87* | *0.04* | *0.09* |  | *0.001* |
|  |  |  |  |  | *Min* | *7* | *47* | *0.02* | *0.05* |  | *0.000* |
|  |  |  |  |  | *Max* | *23* | *459* | *0.08* | *1.87* |  | *0.003* |
|  |  |  |  |  | *Q3* | *19* | *379* | *0.07* | *1.54* |  | *0.003* |
|  | *N. Nor* | *246* | *5721* | *20* | *Median* | *15* | *299* | *0.05* | *1.22* | *146,624* | *0.002* |
|  |  |  |  |  | *Q1* | *13* | *259* | *0.05* | *1.05* |  | *0.002* |
|  |  |  |  |  | *Min* | *7* | *140* | *0.02* | *0.57* |  | *0.001* |
|  |  |  |  |  | *Max* | *23* | *331* | *0.06* | *0.16* |  | *0.002* |
|  |  |  |  |  | *Q3* | *19* | *274* | *0.05* | *0.13* |  | *0.002* |
|  | *C. Nor* | *2128* | *5153* | *14* | *Median* | *15* | *216* | *0.04* | *0.10* | *146,624* | *0.001* |
|  |  |  |  |  | *Q1* | *13* | *187* | *0.04* | *0.09* |  | *0.001* |
|  |  |  |  |  | *Min* | *7* | *101* | *0.02* | *0.05* |  | *0.001* |
|  |  |  |  |  | *Max* | *23* | *834* | *0.20* | *0.39* |  | *0.006* |
|  |  |  |  |  | *Q3* | *19* | *689* | *0.16* | *0.32* |  | *0.005* |
|  | *SW. Nor* | *2128* | *4261* | *36* | *Median* | *15* | *544* | *0.13* | *0.26* | *146,624* | *0.004* |
|  |  |  |  |  | *Q1* | *13* | *471* | *0.11* | *0.22* |  | *0.003* |
|  |  |  |  |  | *Min* | *7* | *254* | *0.06* | *0.12* |  | *0.002* |
|  |  |  |  |  | *Max* | *23* | *1777* | *0.10* | *0.32* |  | *0.012* |
|  |  |  |  |  | *Q3* | *19* | *1468* | *0.08* | *0.27* |  | *0.010* |
| *Total* | *Norway* | *5492* | *17,396* | *77* | *Median* | *15* | *1159* | *0.07* | *0.21* | *146,624* | *0.008* |
|  |  |  |  |  | *Q1* | *13* | *1004* | *0.06* | *0.18* |  | *0.007* |
|  |  |  |  |  | *Min* | *7* | *541* | *0.03* | *0.10* |  | *0.004* |

# 5. Comparative estimates: GROUP1 & GROUP2

## 5.1. The Late Glacial Final Palaeolithic

| 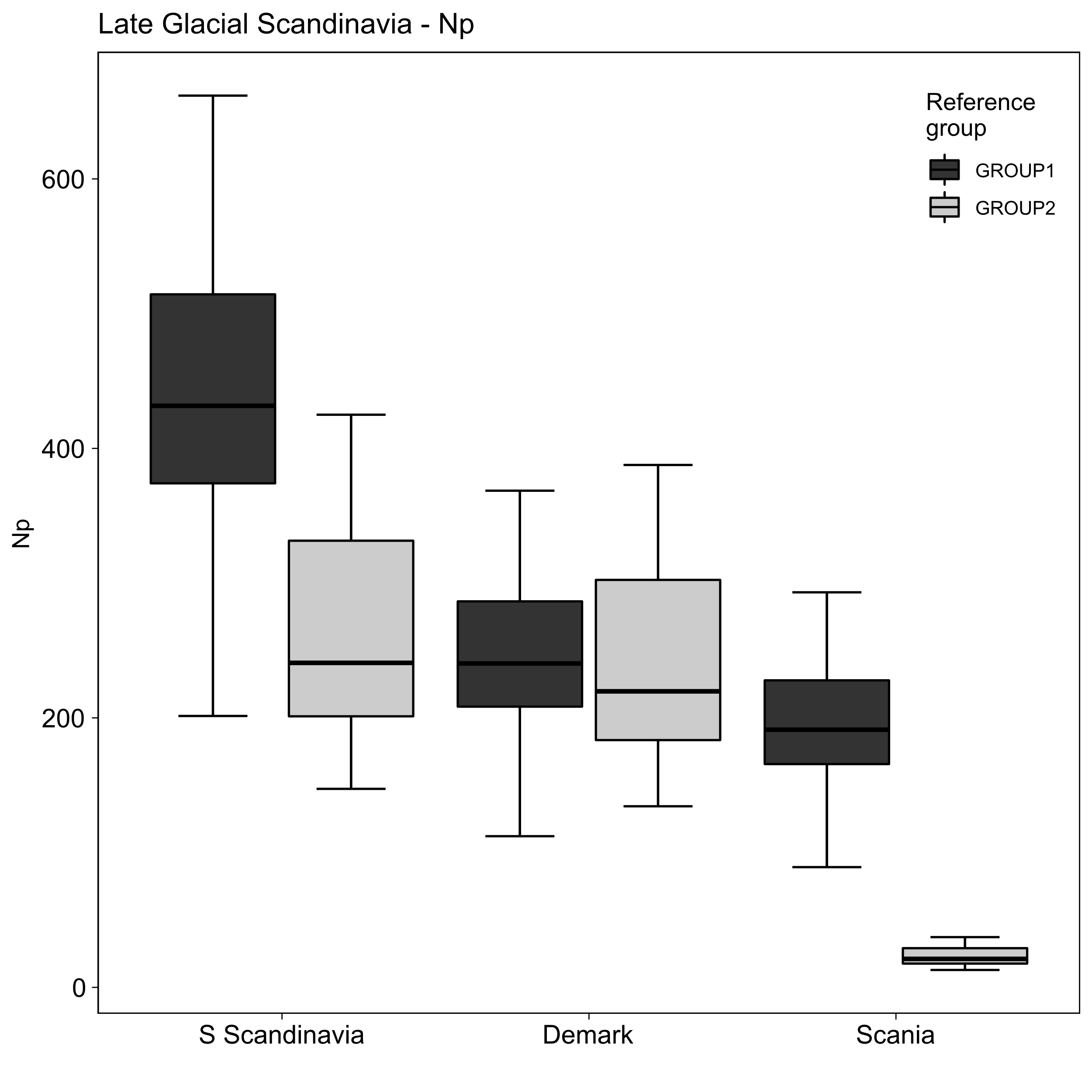 | 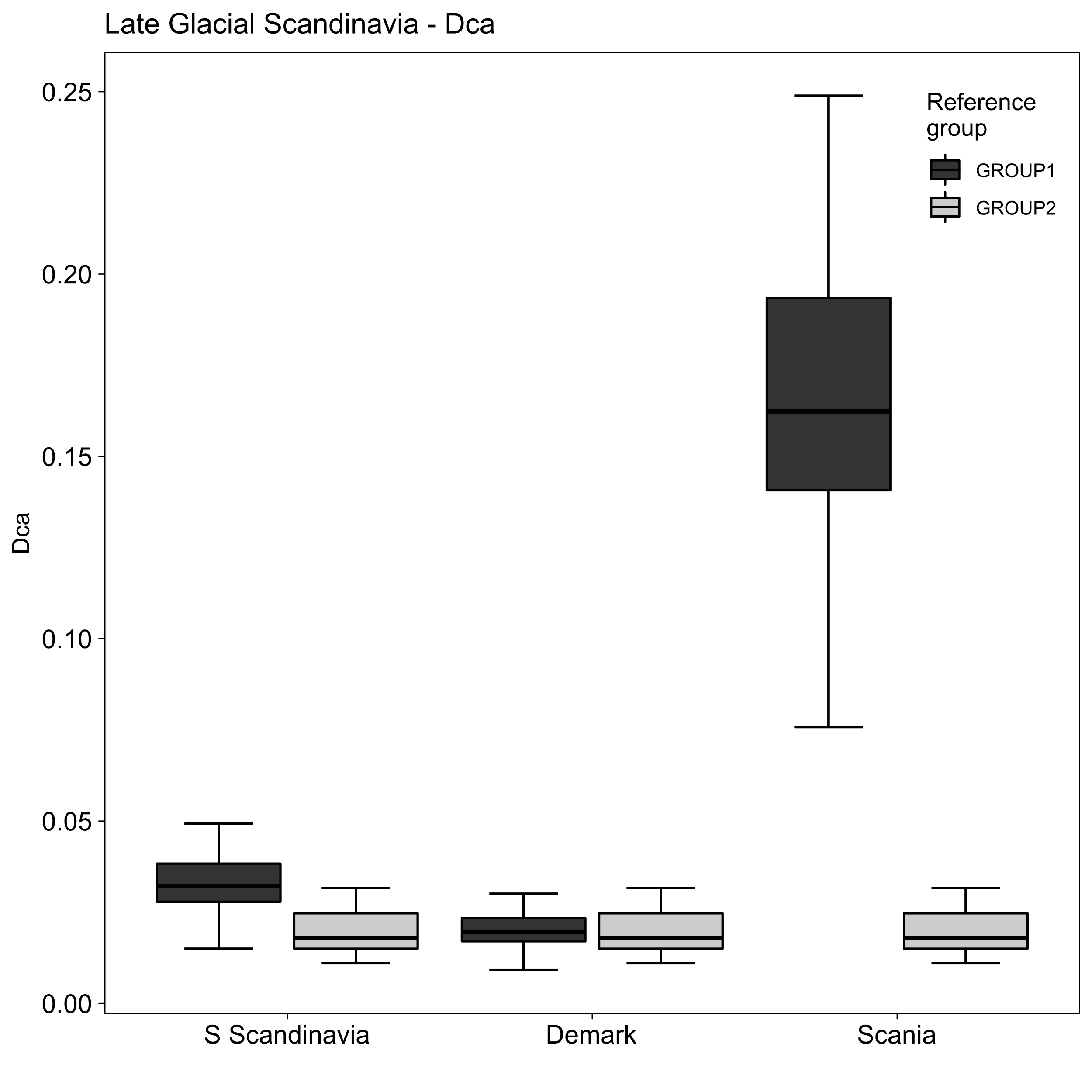 | 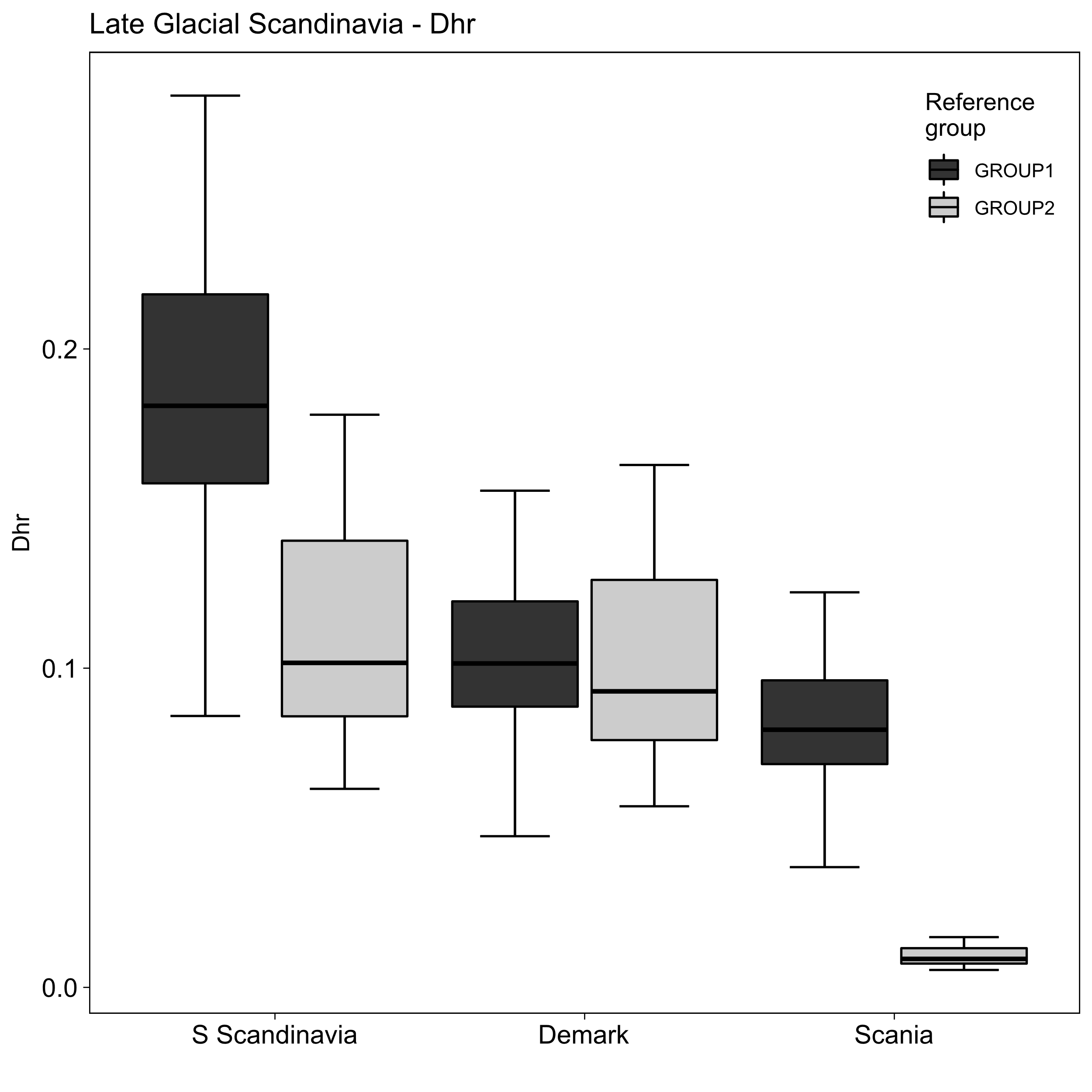 |
| --- | --- | --- |

Figure 7 Box and whisker plots with comparative estimates on total population size (Np) and population densities across core areas (Dca), and home ranges (Dhr), using GROUP1 and GROUP2 social units for predominantly terrestrial hunter-gatherers.

## 5.2. Early Holocene Norway

| 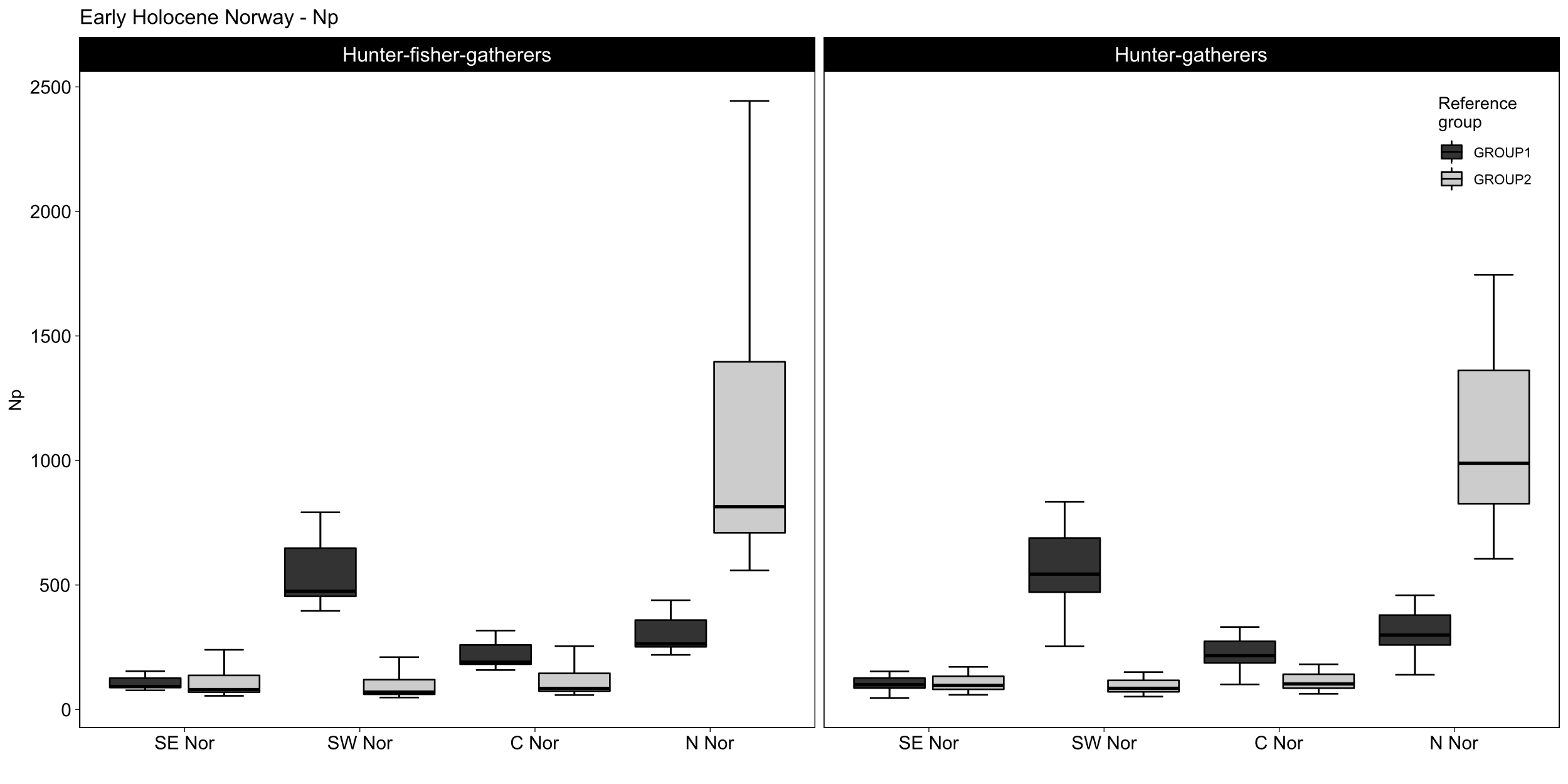 |
| --- |
| 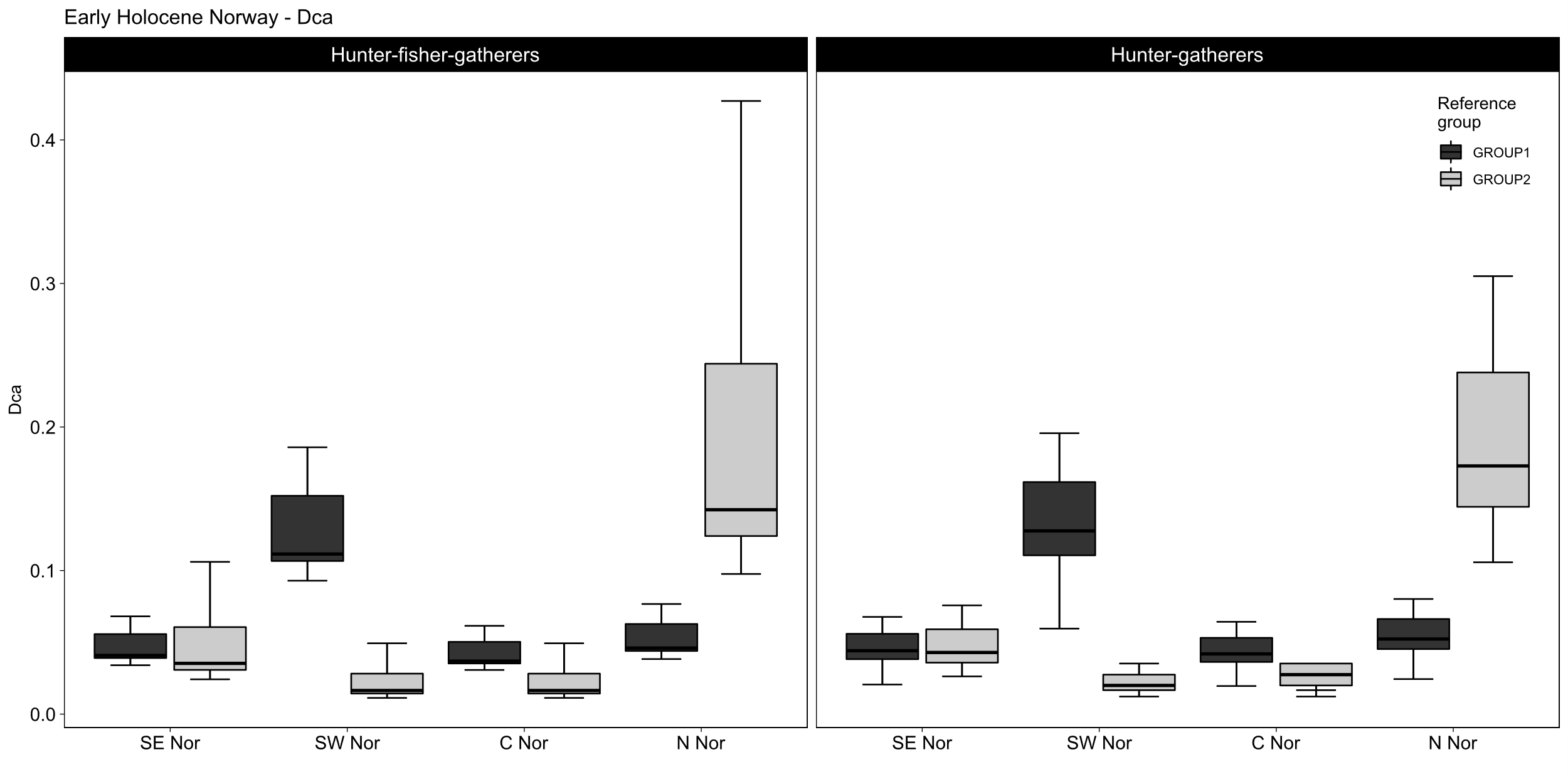 |
| 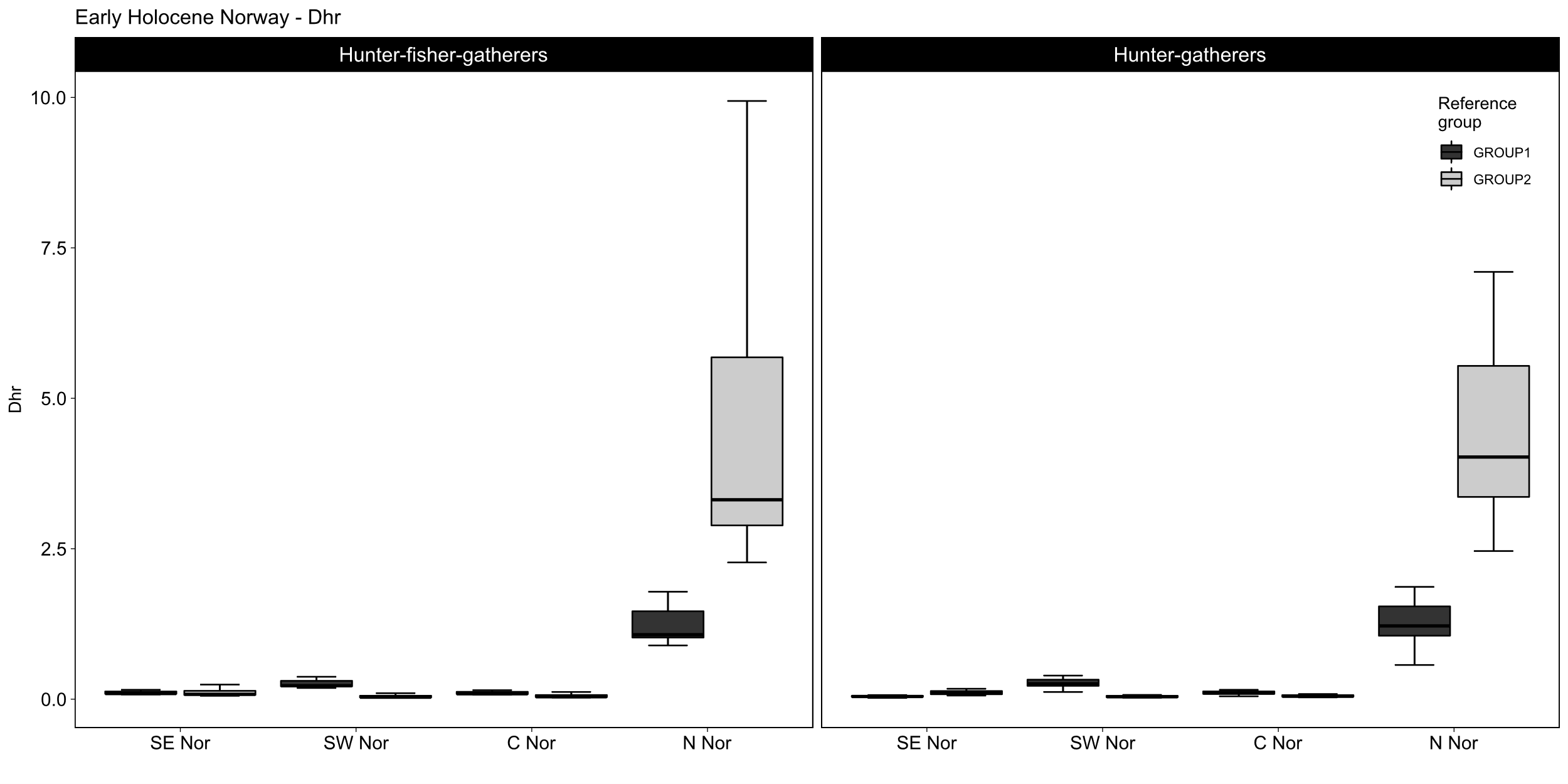 |

Figure 8 Box and whisker plots with comparative estimates on total population size (Np) and population densities across core areas (Dca), and home ranges (Dhr), using both GROUP1 and GROUP2 social units for predominantly marine oriented hunter-gatherers (left panels) and predominantly terrestrial hunter-gatherers (right panels).

# 6. Distribution maps

| 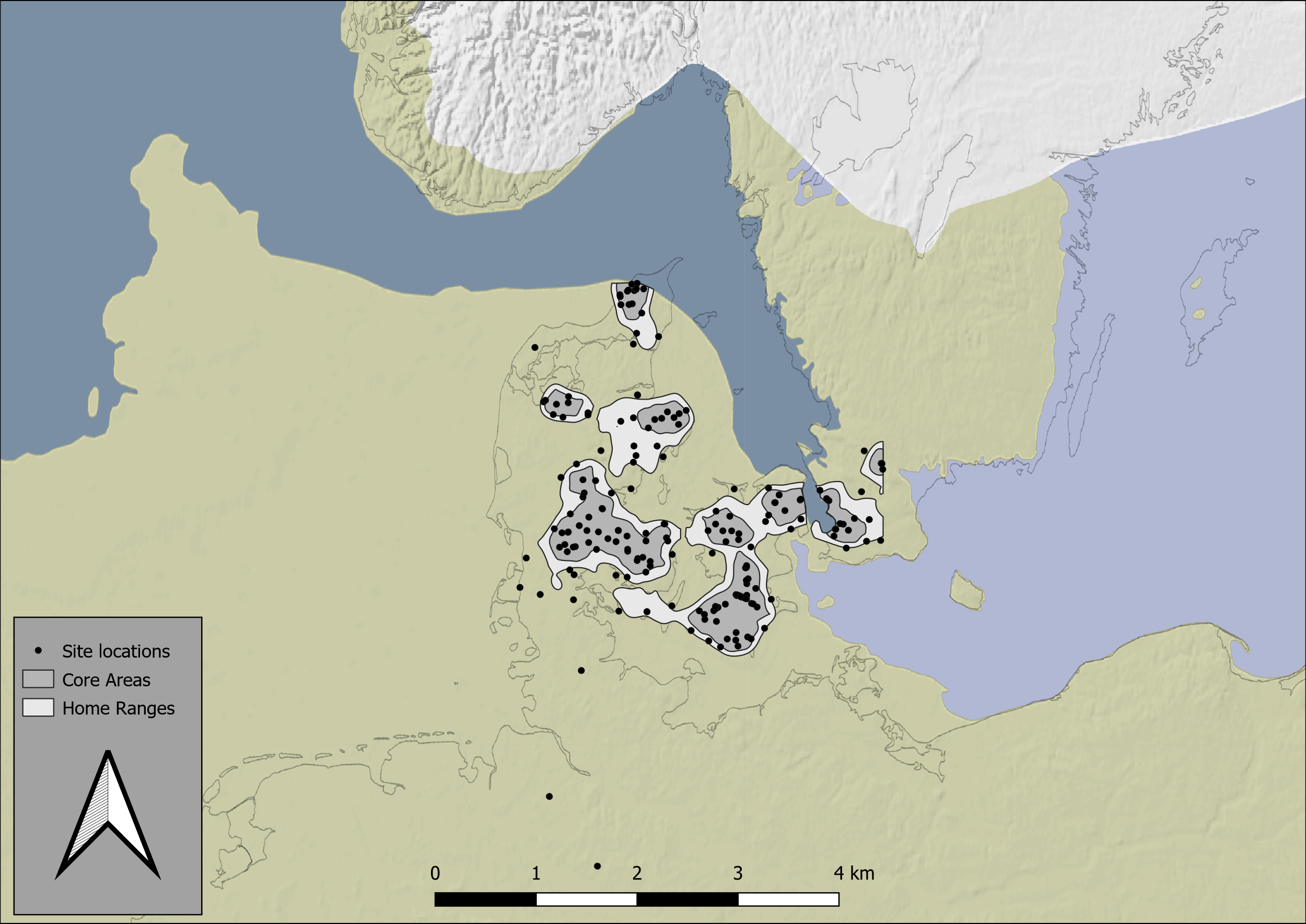 |
| --- |
| 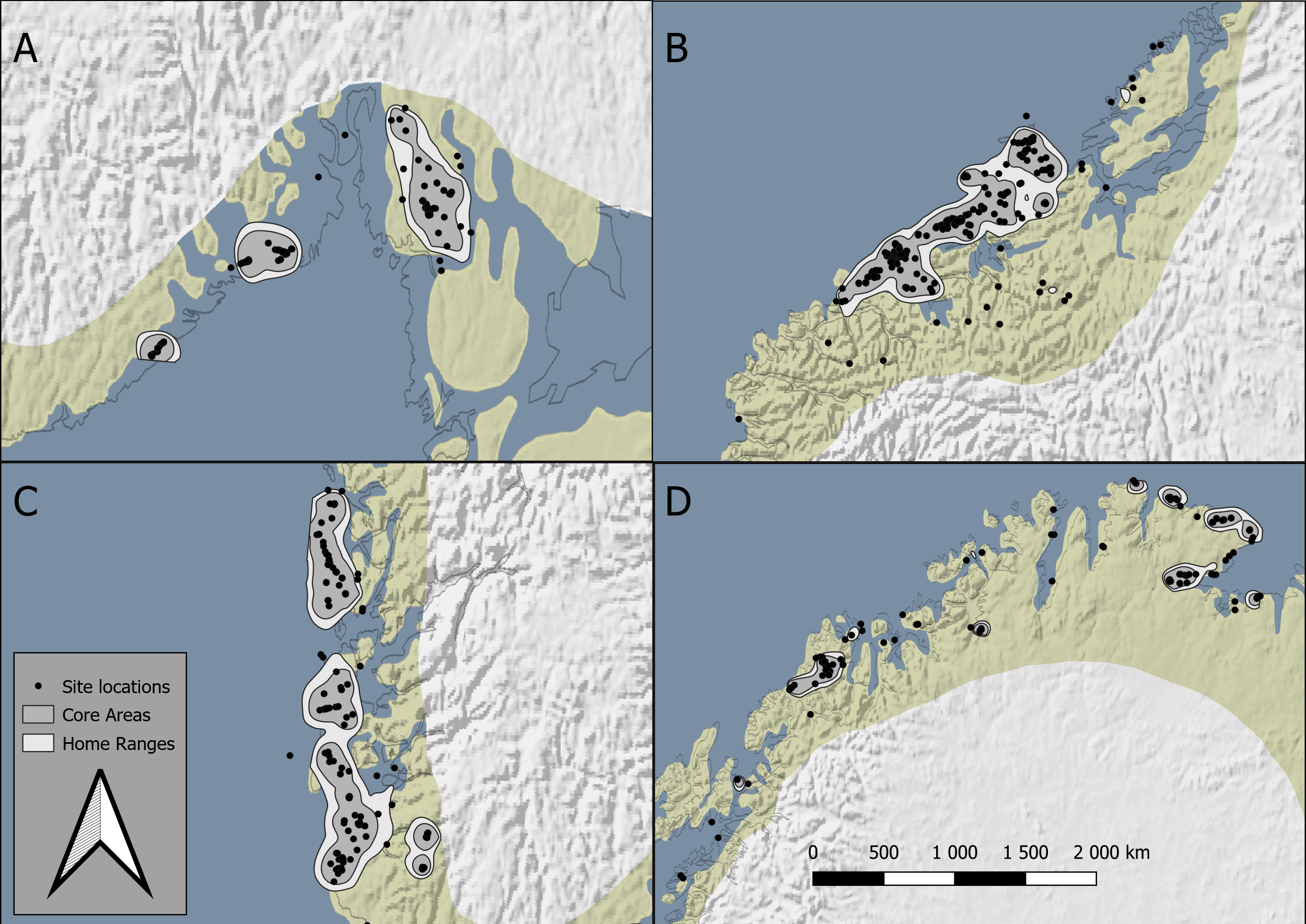 |

Figure 9 Distribution maps of site locations, core areas, and home ranges, from southern Scandinavia during the Late Glacial Final Palaeolithic (top panel) and south-eastern (A), central (B), south-western (C), and northern Norway (D) during the early Holocene (bottom panel). Palaeogeographic maps were compiled by ZBSA after Björck 1995; Brooks et al. 2011; Cohen et al. 2017; Edwards/Brooks 2008; Harff et al. 2017; Lericolais 2017; Moscon et al. 2015; Påsse/Andersson 2005; Seguinot et al. 2018; Stroeven et al. 2016; Subetto et al. 2017; Weaver et al. 2003. Projection: UTM32N; EPSG: 25832.

# 7. Literature

Björck, S., 1995. A review of the history of the Baltic Sea, 13.0-8.0 ka BP. Quaternary International. 27, 19–40. https://doi.org/10.1016/1040-6182(94)00057-C

Anthony J. Brooks, Sarah L. Bradley, Robin J. Edwards & Nicola Goodwyn (2011) The palaeogeography of Northwest Europe during the last 20,000 years, Journal of Maps, 7:1, 573-587, DOI: [10.4113/jom.2011.1160](https://doi.org/10.4113/jom.2011.1160)

Boethius, A., Ahlström, T., 2018. Fish and resilience among Early Holocene foragers of southern Scandinavia: A fusion of stable isotopes and zooarchaeology through Bayesian mixing modelling. *Journal of Archaeological Science*. 93, 196–210. https://doi.org/10.1016/j.jas.2018.02.018

Cohen, K.M., Westley, K., Erkens, G., Hijma, M.P., Weerts, H.J.T., 2017. The North Sea, in: Flemming, N.C., Harff, J., Moura, D., Burgess, A., Bailey, G.N. (Eds.), Submerged Landscapes of the European Continental Shelf. John Wiley & Sons, Ltd, Oxford, UK, pp. 147–186. https://doi.org/10.1002/9781118927823.ch7

Edwards, R.J., Brooks, A.J. (2008) The Island of Ireland: Drowning the Myth of an Irish Land-bridge? In: Davenport, J.J., Sleeman, D.P., Woodman, P.C. (eds.) Mind the Gap: Postglacial Colonisation of Ireland. Special Supplement to The Irish Naturalists’ Journal. pp 19-34

Harff, J., Flemming, N.C., Groh, A., Hünicke, B., Lericolais, G., Meschede, M., Rosentau, A., Sakellariou, D., Uścinowicz, S., Zhang, W., Zorita, E., 2017. Sea Level and Climate, in: Flemming, N.C., Harff, J., Moura, D., Burgess, A., Bailey, G.N. (Eds.), Submerged Landscapes of the European Continental Shelf. John Wiley & Sons, Ltd, Oxford, UK, pp. 11–49. https://doi.org/10.1002/9781118927823.ch2

Isaaks, E. and Srivastava, R., 1990. *Applied Geostatistics*. New York: Oxford University Press.

Kretschmer, I., 2015. Demographische Untersuchungen zu Bevölkerungsdichten, Mobilität und Landnutzungsmustern im späten Jungpaläolithikum, Kölner Studien zur prähistorischen Archäologie. VML Verlag Marie Leidorf GmbH, Rahden/Westf.

Lericolais, G., 2017. Late Pleistocene Environmental Factors defining the Black Sea, and Submerged Landscapes on the Western Continental Shelf, in: Flemming, N.C., Harff, J., Moura, D., Burgess, A., Bailey, G.N. (Eds.), Submerged Landscapes of the European Continental Shelf. John Wiley & Sons, Ltd, Oxford, UK, pp. 479–495. https://doi.org/10.1002/9781118927823.ch17

Lundström, V., Riede, F., 2019. A spatially explicit model of Final Palaeolithic population densities for southern Scandinavia in the period between 14,000 and 12,700 cal BP. J. Archaeol. Sci. Rep. 26, 101886. https://doi.org/10.1016/j.jasrep.2019.101886

Moscon, G., Corregiari, A., Stefani, C., Fontana, A. and Remia, A. 2015. Very-high resolution analysis of a transgessive deposit in the Northern Adriactic sea (Italy). Alpine and Mediterranean Quaternary 28, pp. 121-129.

Pasanen, A., Lunkka, J.P., Putkinen, N., 2010. Reconstruction of the White Sea Basin during the late Younger Dryas. Boreas 39, pp. 273–285. https://doi.org/10.1111/j.1502-3885.2009.00128.x

Patton, H., Hubbard, A., Andreassen, K., Auriac, A., Whitehouse, P.L., Stroeven, A.P., Shackleton, C., Winsborrow, M., Heyman, J., Hall, A.M., 2017. Deglaciation of the Eurasian ice sheet complex. Quat. Sci. Rev. 169, pp. 148–172. https://doi.org/10.1016/j.quascirev.2017.05.019

Påsse, T., Andersson, L., 2005. Shore-level displacement in Fennoscandia calculated from empirical data. GFF 127, pp. 253–268. <https://doi.org/10.1080/11035890501274253>

Riede, F., Hoggard, C., Shennan, S., 2019. Reconciling material cultures in archaeology with genetic data requires robust cultural evolutionary taxonomies. Palgrave Commun. 5. https://doi.org/10.1057/s41599-019-0260-7

Seguinot, J., Ivy-Ochs, S., Jouvet, G., Huss, M., Funk, M., Preusser, F., 2018. Modelling last glacial cycle ice dynamics in the Alps. The Cryosphere 12, pp. 3265–3285. <https://doi.org/10.5194/tc-12-3265-2018>

Stroeven, A.P., Hättestrand, C., Kleman, J., Heyman, J., Fabel, D., Fredin, O., Goodfellow, B.W., Harbor, J.M., Jansen, J.D., Olsen, L., Caffee, M.W., Fink, D., Lundqvist, J., Rosqvist, G.C., Strömberg, B., Jansson, K.N., 2016. Deglaciation of Fennoscandia. Quat. Sci. Rev. 147, pp. 91–121. <https://doi.org/10.1016/j.quascirev.2015.09.016>

Subetto, D., Zobkov, M., Potakhin, M., Tarasov, A., 2017. Paleoreconstructions of Lake Onego. Development in the Late Pleistocene and Holocene. URL https://www.arcgis.com/apps/MapJournal/index.html?appid=47d76ba2004e463d96eba1d8a1825fe1

Weaver, A.J., 2003. Meltwater Pulse 1A from Antarctica as a Trigger of the Bolling-Allerod Warm Interval. Science 299, pp. 1709–1713. https://doi.org/10.1126/science.1081002
